# Crystal structures of zinc(II) coordination complexes with iso­quinoline *N*-oxide

**DOI:** 10.1107/S2056989025000180

**Published:** 2025-01-14

**Authors:** Erin N. Groneck, Nathan Peek, Will E. Lynch, Clifford W. Padgett

**Affiliations:** a Department of Biochemistry Chemistry and Physics Georgia Southern University, Armstrong Campus 11935 Abercorn Street Savannah GA 31419 USA; University of Aberdeen, United Kingdom

**Keywords:** crystal structure, zinc(II) coordination complex, iso­quinoline *N*-oxide, Hirshfeld surface analysis

## Abstract

The reaction of one equivalent of zinc(II) halide salts with two equivalents of iso­quinoline *N*-oxide (iQNO; C_9_H_7_NO) in methanol yields compounds of the general formula [Zn*X*_2_(iQNO)_2_], with *X* = Cl^−^ (**I**), Br^−^ (**II**) and I^−^ (**III**). However, starting with zinc(II) perchlorate or nitrate leads to the formation of complex ions with the compositions [Zn(iQNO)_6_](*X*)_2_ for *X* = ClO_4_^−^ (**IV**), and [Zn(iQNO)(H_2_O)_5_](iQNO)_2_*X*_2_ for *X* = NO_3_^−^ (**V**). Complexes (**I**), (**II**) and (**III**), namely di­chlorido­bis­(iso­quinoline *N*-oxide-κ*O*)zinc(II) [ZnCl_2_(C_9_H_7_NO)_2_], di­bromido­bis­(iso­quinoline *N*-oxide-κ*O*)zinc(II) [ZnBr_2_(C_9_H_7_NO)_2_], and di­iodido­bis­(iso­quinoline *N*-oxide-κ*O*)zinc(II) [ZnI_2_(C_9_H_7_NO)_2_], each exhibit a distorted tetra­hedral coordination geometry around the zinc(II) ion coordinated by two iQNO ligands bound through the oxygen atom and two halide ions. The zinc ion lies on a crystallographic twofold axis in the bromo complex. The *X*—Zn—*X* bond angles are approximately 15–17° larger than the O—Zn—O bond angles resulting in the observed tetra­hedral distortion. In complex (**IV**), hexa­kis­(iso­quinoline *N*-oxide-κ*O*)zinc(II) bis­(perchlorate), [Zn(C_9_H_7_NO)_6_](ClO_4_)_2_, the zinc(II) ion occupies a special position with 3 site symmetry and is octa­hedrally coordinated by six iQNO ligands, albeit with slight distortions evidenced by a spread of *cis* bond angles from 85.82 (4) to 94.18 (4)°. The chlorine atom of the perchlorate anion lies on a crystallographic threefold axis. Finally, complex (**V**) crystallizes with a pseudo-octa­hedral geometry; penta­aqua­(iso­quinoline *N*-oxide-κ*O*)zinc(II) dinitrate–iso­quinoline *N*-oxide (1/2), [Zn(C_9_H_7_NO)(H_2_O)_5_](NO_3_)_2_·2(C_9_H_7_NO). The nitrate ions and non-coordin­ated iQNO mol­ecules engage in π-stacking and hydrogen-bonding inter­actions with the coordinated water mol­ecules. The iQNO—Zn—O equatorial bond angles range from 88.98 (9) to 94.90 (9)°, with the largest deviation from a perfect octa­hedral angle attributed to the influence of a weak C—H⋯O (from water) inter­action (2.287 Å) involving the bound iQNO ligand.

## Abstract 2

The structures of five related zinc coordination compounds with iso­quinoline *N*-oxide and other ligands or counter-ions are presented.

## 1. Chemical context

There is a great deal of inter­est in the chemistry of *N*-oxides due to their ubiquity in nature, recent advances in pharmaceutical chemistry (see, for example, Kobus *et al.*, 2024 ▸), and their important roles in synthesis and materials science (*e.g.*, Ang *et al.*, 2024 ▸; Larin & Fershtat, 2022 ▸). Functional features of importance include the highly polar N—O bond, which is capable of forming strong inter­actions with cations. Aromatic *N*-oxides are more stable and have a slightly higher bond order than their aliphatic counterparts, as they allow for back-donation of electron density into the π* orbital (Lukomska *et al.*, 2015 ▸; Greenberg *et al.*, 2020 ▸). Recently, the effect of iso­quinoline­quinone *N*-oxides as potent anti­cancer agents has also been reported (Kruschel *et al.*, 2024 ▸).

Transformations involving *N*-oxides and transition metals include both the synthesis and reactivity of these complexes (see, for example, Eppenson, 2003 ▸; Moustafa *et al.*, 2014 ▸). These transformations take advantage of the Lewis acid/base properties of metals and the polar *N*-oxide ligands. Owing to this, there is considerable inter­est in metal complexes that bind *N*-oxides and their structures. We have previously reported the structures of zinc(II) halide complexes with quinoline *N*-oxide (QNO) (Padgett *et al.*, 2022 ▸). In the present study, we extend our work on QNO zinc complexes to iso­quinoline *N*-oxide (iQNO) complexes. Herein, we report five iQNO/zinc(II) complexes containing chloride, bromide, iodide, perchlorate, and nitrate anions.
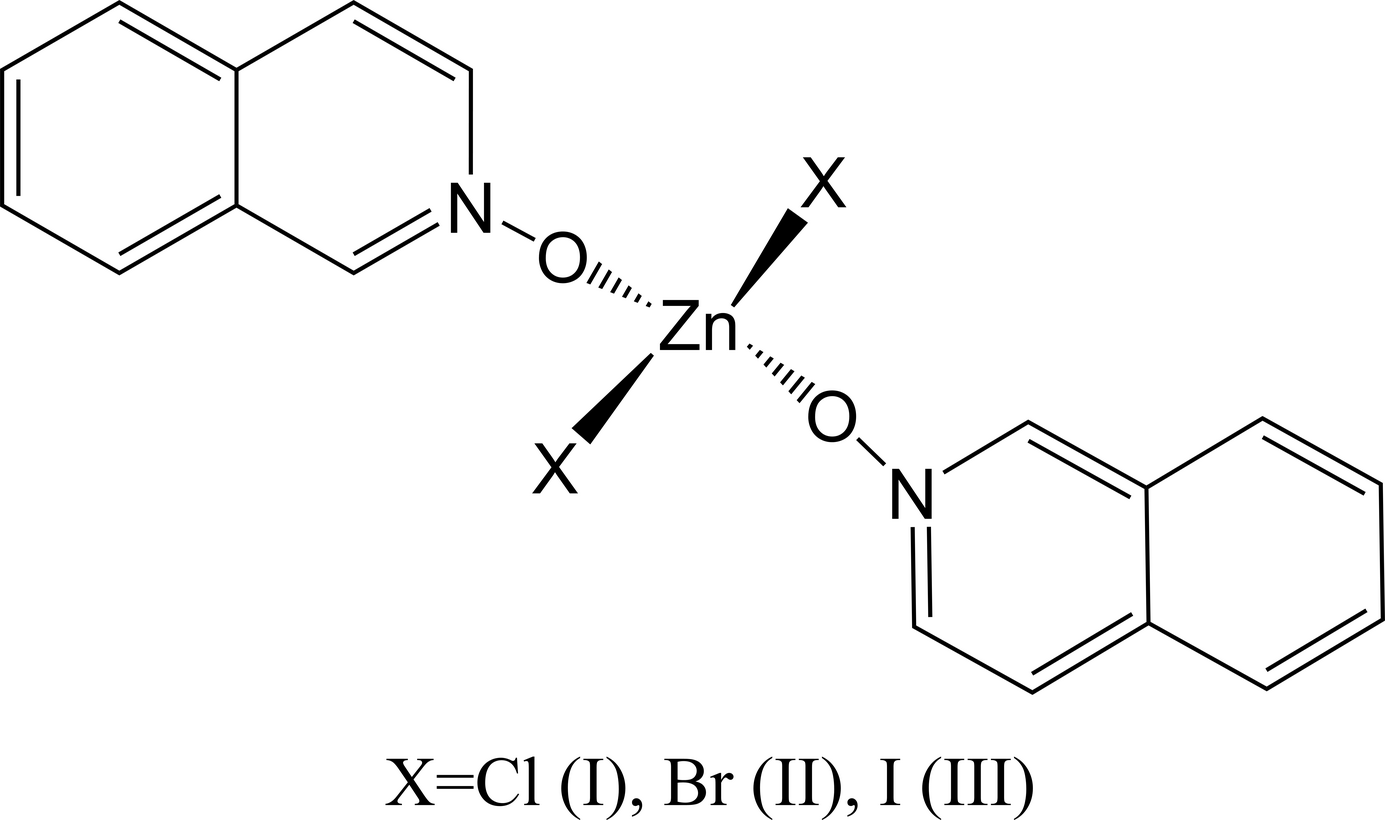

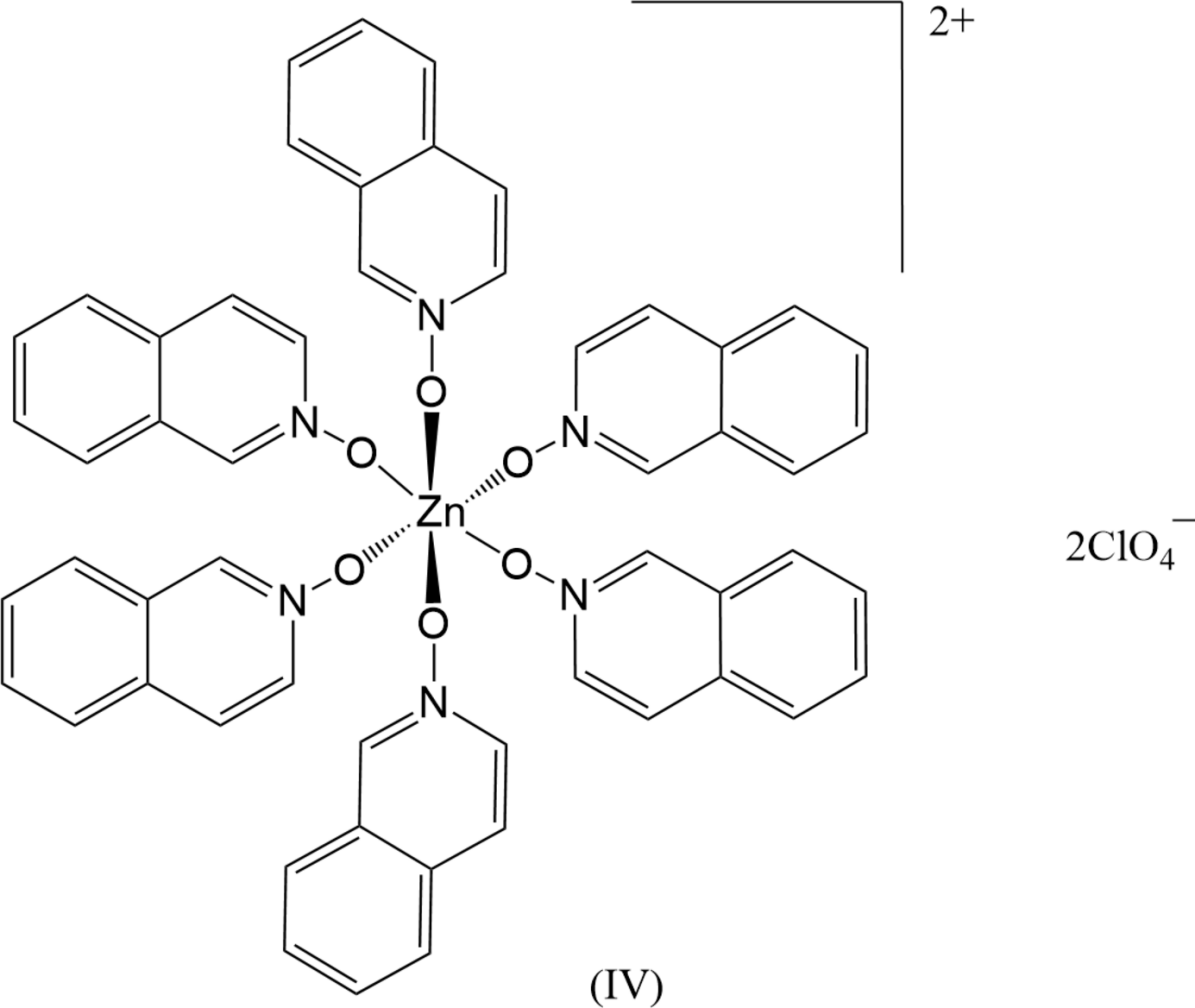

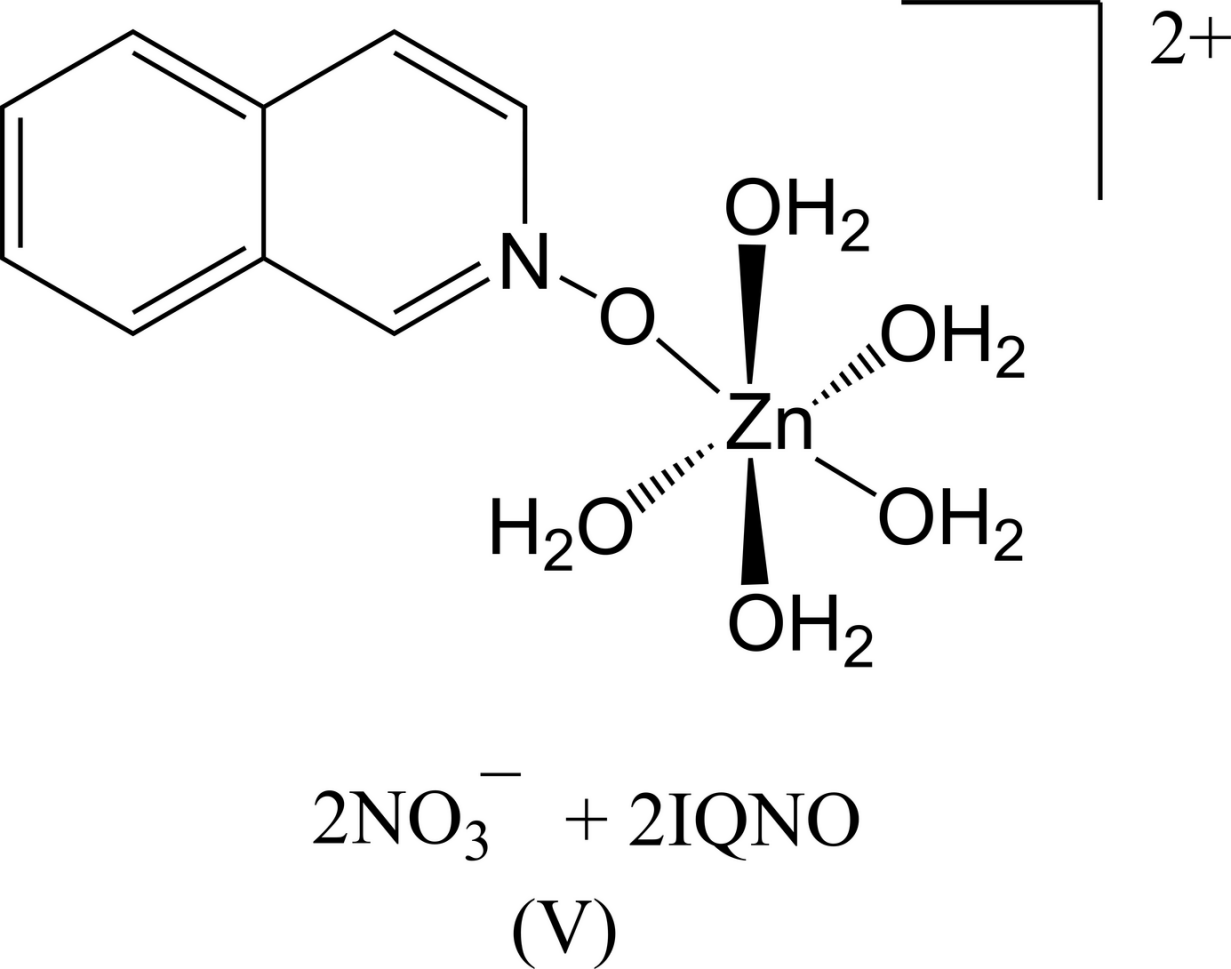


The three zinc(II) halide complexes can be formulated as mononuclear Zn(X)_2_(iQNO)_2_ species in a distorted tetra­hedral environment. The non-coordinating perchlorate and nitrate derivatives yield significantly different complexes. The perchlorate complex is hexa­coordinated, with six iQNO mol­ecules bound to the metal ion in a pseudo-octa­hedral environment, formulated as [Zn(iQNO)_6_](ClO_4_)_2_. The nitrate derivative is also six-coordinate but features five water mol­ecules and one iQNO ligand in the coordination sphere, with two π-stacked iQNOs and two nitrate anions present in the structure.

## 2. Structural commentary

**Figure 1:**
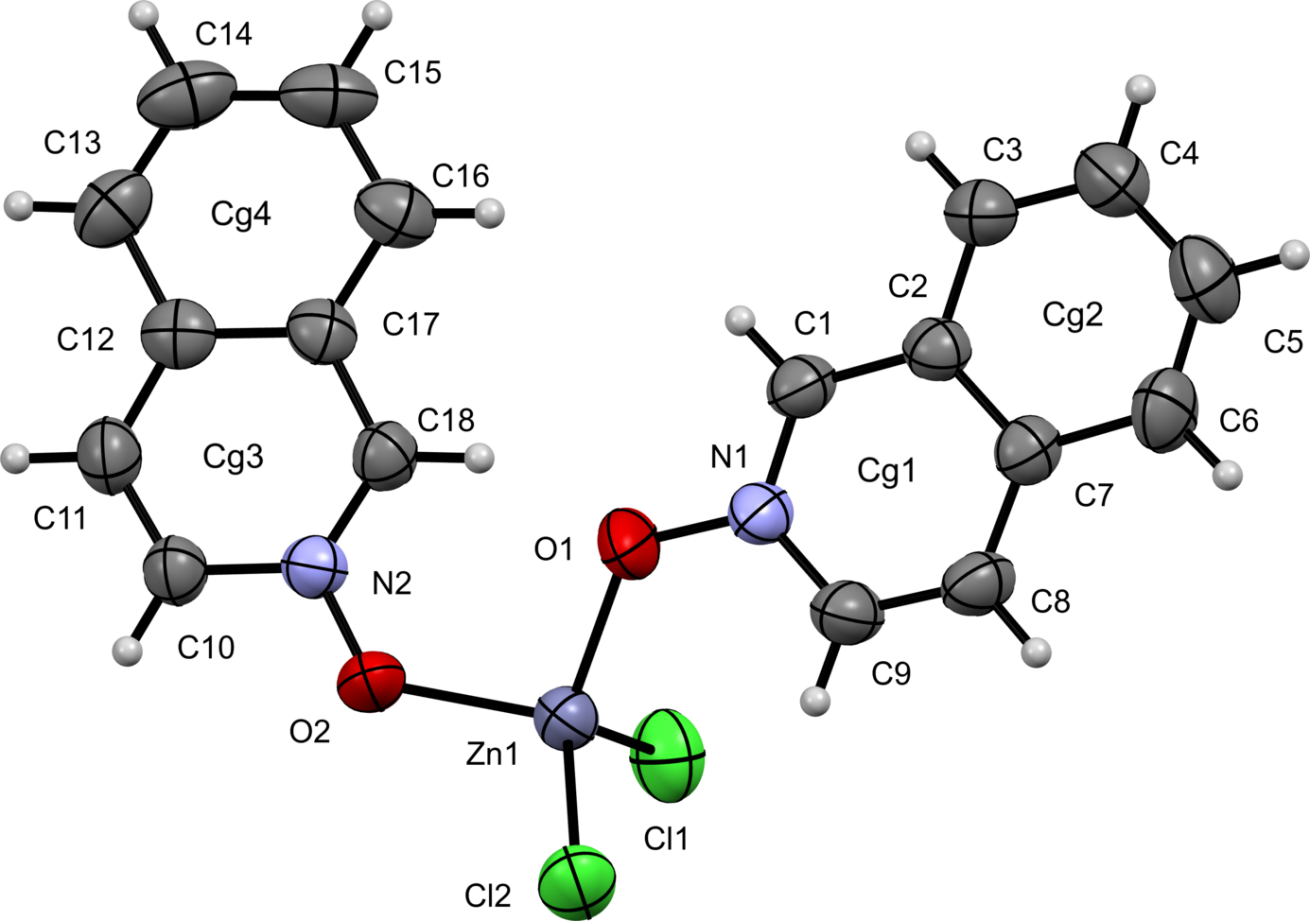
The mol­ecular structure of (**I**) with displacement ellipsoids drawn at the 50% probability level.

Compound (**I**) crystallizes in the triclinic space group *P*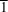 (Fig. 1 ▸) and exhibits a distorted tetra­hedral coordination environment around the Zn center. The Cl—Zn—Cl bond angle is 117.35 (6)° and the O—Zn—O angle is 101.78 (13)°. The Zn—O bond distances are 1.999 (3) Å (Zn1—O1) and 1.968 (3) Å (Zn1—O2), while the Zn—Cl bond distances are 2.2088 (14) Å (Zn1—Cl1) and 2.2147 (13) Å (Zn1—Cl2).

**Figure 2:**
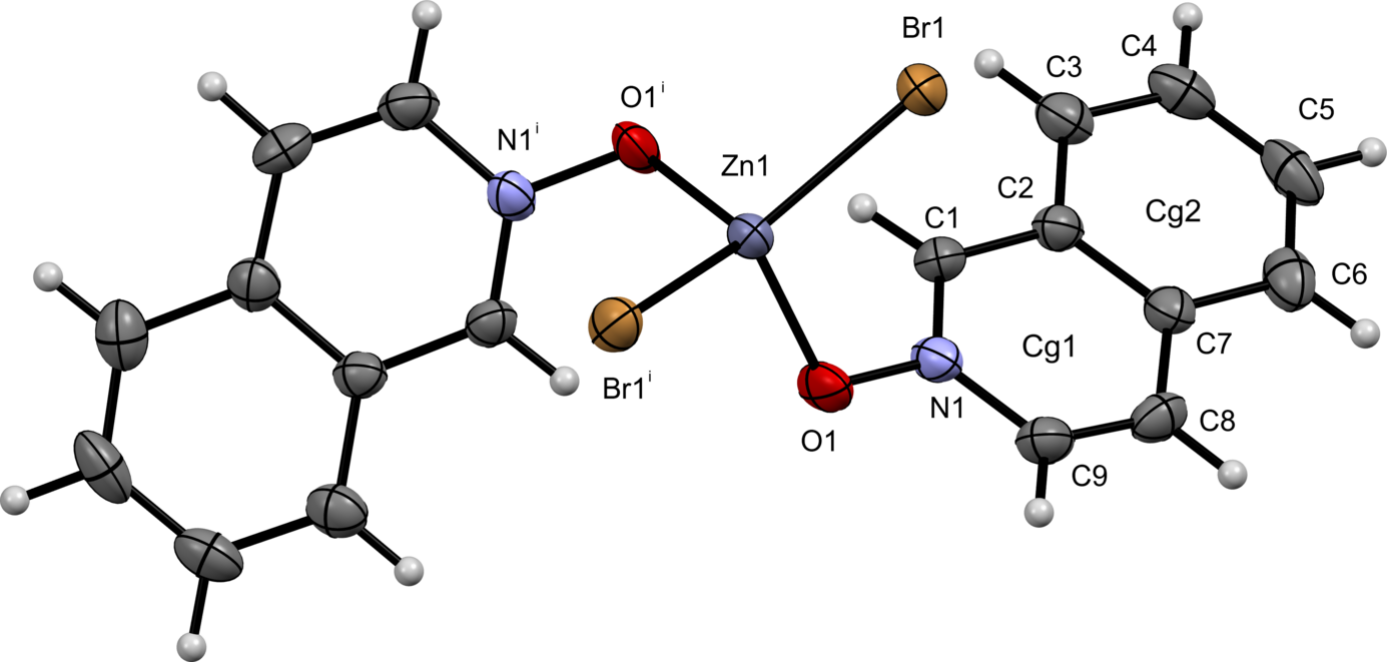
The mol­ecular structure of (**II**) with displacement ellipsoids drawn at the 50% probability level.

The bromide analog, complex (**II**), crystallizes in the monoclinic space group *C*2/*c*, with the zinc ion lying on a crystallographic twofold axis. The Zn1—Br1 bond distance is 2.3476 (7) Å, whereas the Zn1—O1 bond distance is 1.995 (4) Å. The Br1—Zn1—Br1^i^ [symmetry code: (i) 1 − *x*, *y*, 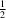 − *z*) bond angle is more open at 119.21 (5)° compared to the O1—Zn1—O1^i^ bond angle of 101.6 (3)° in the pseudo-tetra­hedral environment (Fig. 2 ▸).

**Figure 3:**
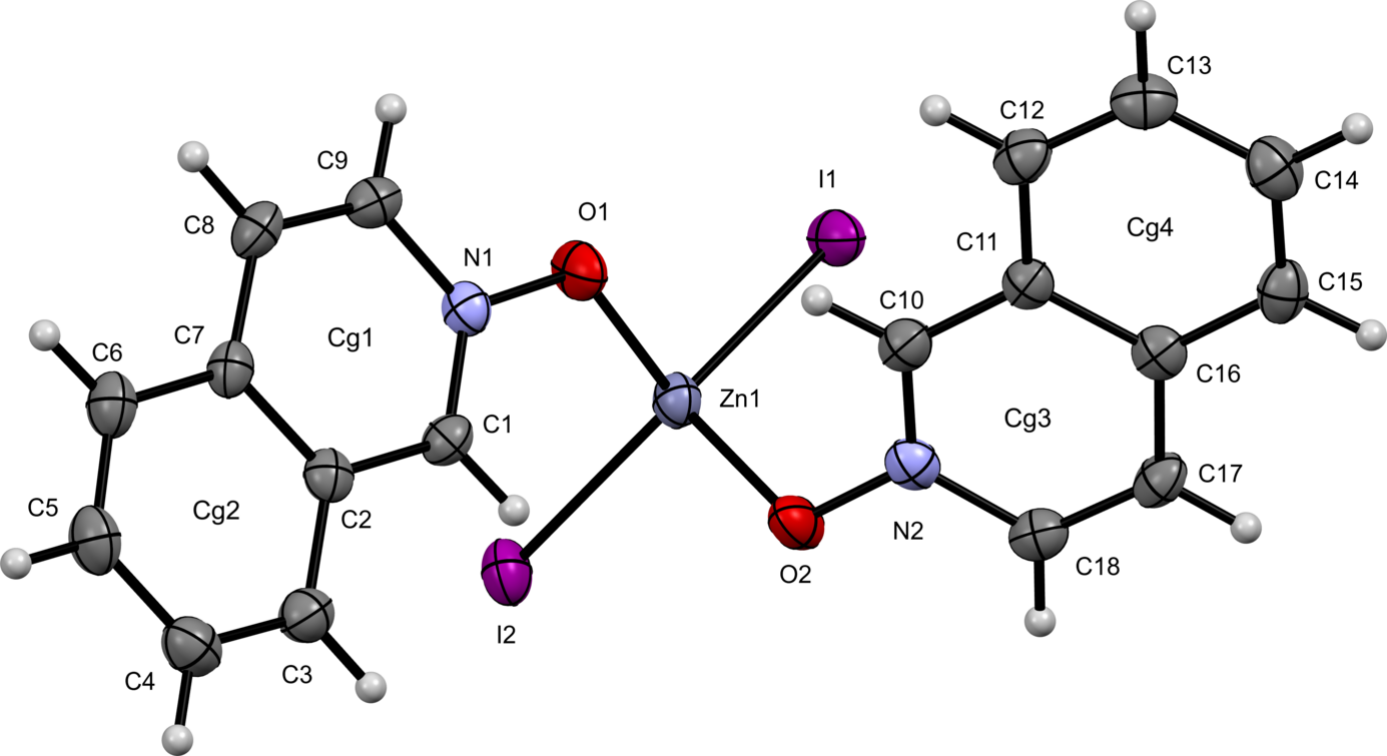
The mol­ecular structure of (**III**) with displacement ellipsoids drawn at the 50% probability level.

For the iodide derivative, complex (**III**), which crystallizes in the triclinic space group *P*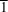 (Fig. 3 ▸), the pseudo-tetra­hedral coordination environment seen in (**I**) is preserved. The I1—Zn1—I2 bond angle is even more open at 122.378 (14)°, with Zn1—I1 and Zn1—I2 bond distances of 2.5591 (4) Å and 2.5504 (4) Å, respectively. The O1—Zn1—O2 bond angle is compressed at 103.62 (9)°, with Zn1—O1 and Zn1—O2 bond distances of 2.0130 (19) Å and 2.016 (2) Å, respectively.

**Figure 4:**
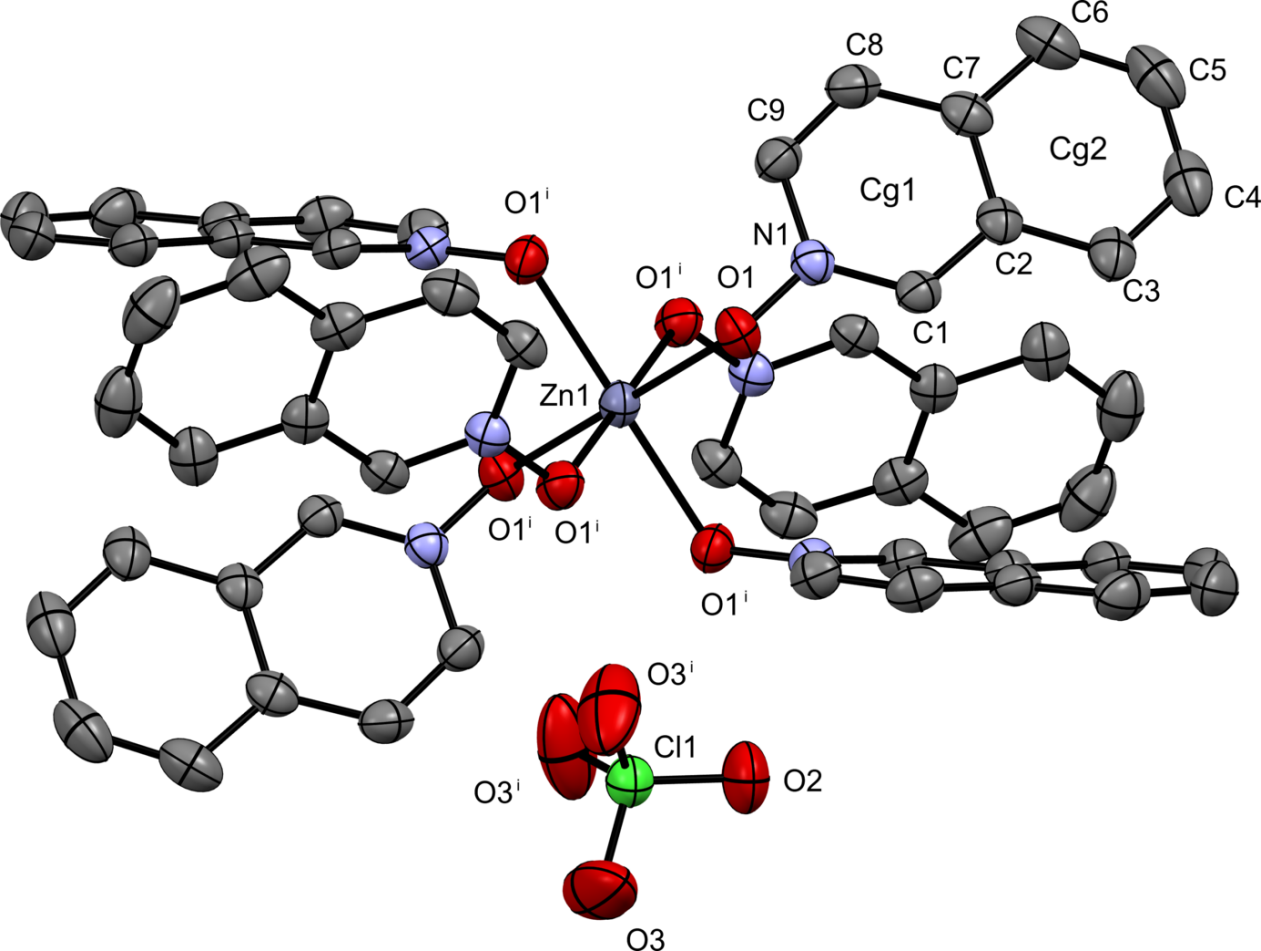
The mol­ecular structure of (**IV**) with displacement ellipsoids drawn at the 50% probability level. Hydrogen atoms removed for clarity.

Complex (**IV**), the perchlorate derivative, crystallizes in the trigonal space group *R*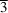 (Fig. 4 ▸) and adopts a pseudo-octa­hedral arrangement around the Zn^II^ center, coordinated by six iQNO mol­ecules. Two perchlorate ions reside in the lattice. The O1—Zn1—O1′ bond angles range from 85.82 (4) to 94.18 (4)°, and the associated Zn1—O1 bond distances are 2.1008 (11) Å.

**Figure 5:**
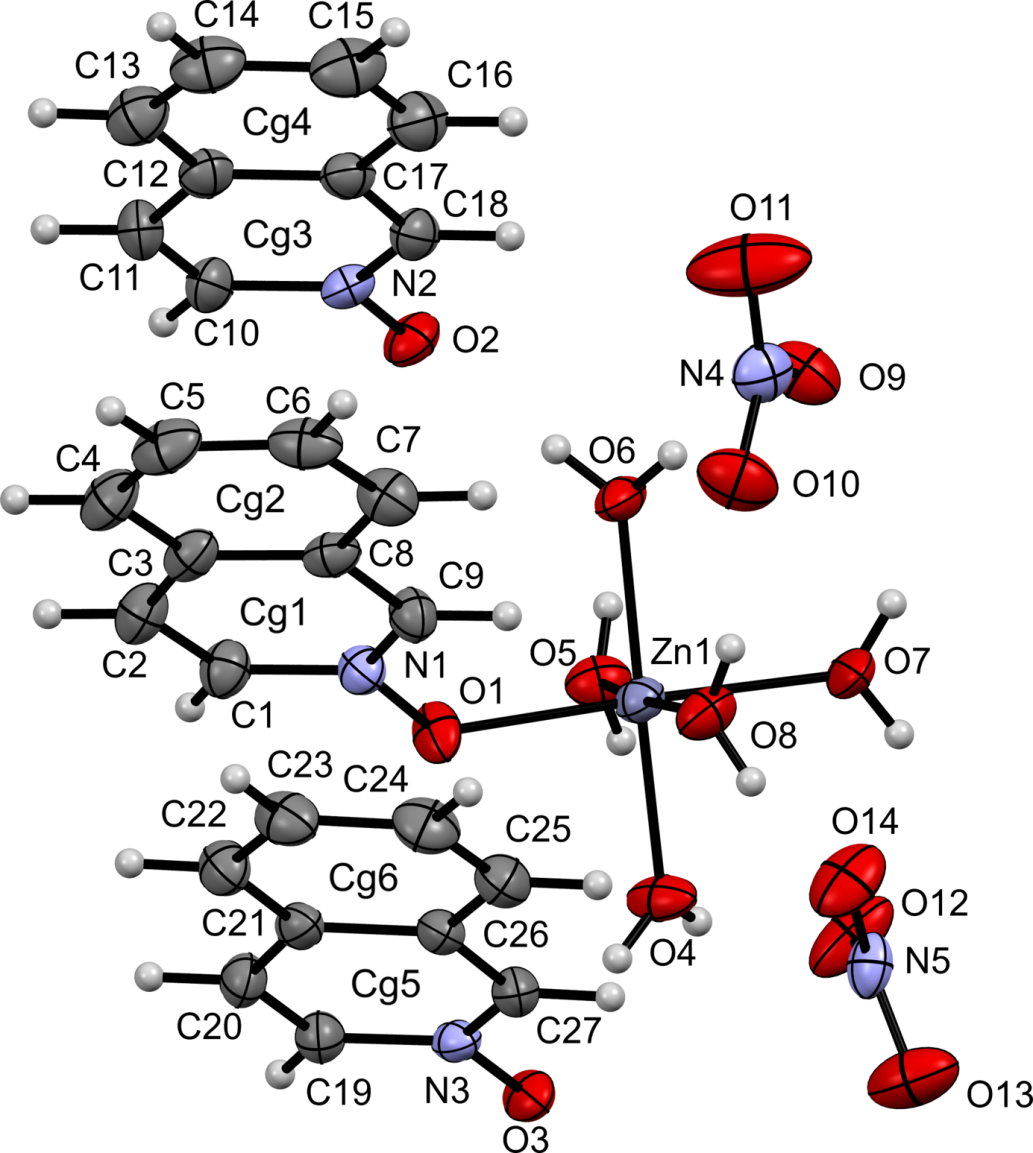
The mol­ecular structure of (**V**) with displacement ellipsoids drawn at the 50% probability level.

Compound (**V**) crystallizes in the triclinic space group *P*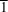 and exhibits a pseudo-octa­hedral arrangement around the Zn^II^ center (Fig. 5 ▸). Of the five water mol­ecules coordinated to the zinc ion, the equatorial Zn1—O bond distances range from 2.015 (2) Å to 2.130 (2) Å, while the axial Zn1—O7 bond distance is slightly longer at 2.174 (2) Å. The iQNO ligand is coordinated *via* O1 [Zn1—O1 = 2.1078 (19) Å], a distance comparable to the Zn—O bonds to water.

The coordinated iQNO ligands participate in π–π stacking inter­actions. The centroid-to-centroid distances between aromatic rings lie in the range of approximately 3.66–3.97 Å. Hydrogen bonding in compound (**V**) is also notable. The nitrate ion associated with N4 (bearing atoms O9, O10 and O11) accepts hydrogen bonds from water mol­ecules O6 [O6⋯O9 = 2.723 (3) Å] and O8 [O8⋯O10 = 2.808 (3) Å]. Similarly, the nitrate ion associated with N5 (O12, O13, O14) accepts a hydrogen bond from O8 [O8⋯O12 = 2.710 (3) Å]. The iQNO ligands also participate in hydrogen bonding: O2 and O3 from the iQNO moieties accept hydrogen bonds from O6 and O4, respectively [O6⋯O2 = 2.651 (3) Å; O4⋯O3 = 2.634 (3) Å] (Table 1 ▸).

## 3. Supra­molecular features

**Figure 6:**
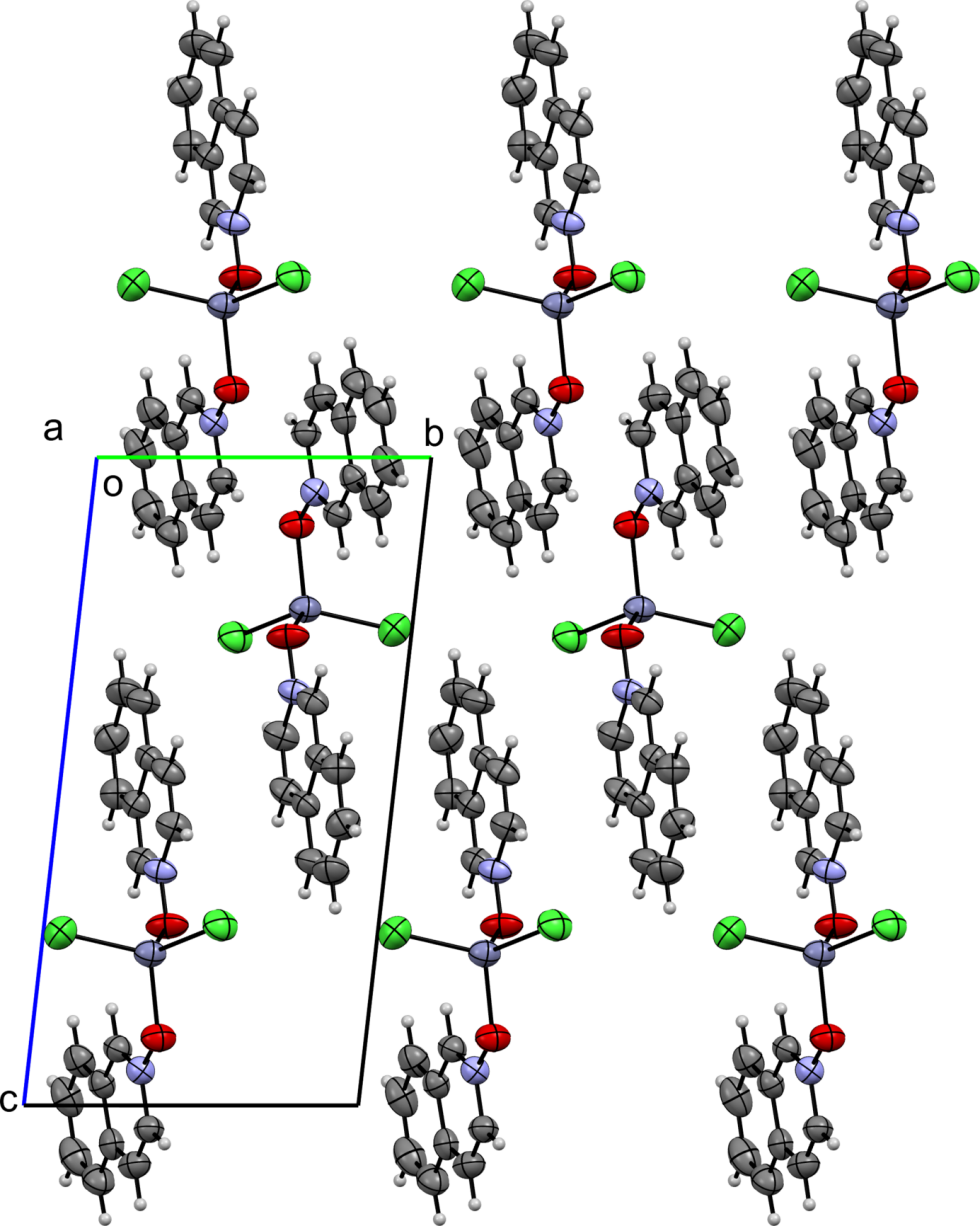
A view along the *a-*axis direction of the crystal packing of (**I**).

Figs. 6 ▸–10 ▸ ▸ ▸ ▸ show the crystal packing of compounds (**I**)–(**V**), respectively. In the packing of (**I**), (**II**), (**III**), and (**IV**) the packing is consolidated by van der Waals inter­actions and π–π stacking. In the case of (**V**), there is an additional network of inter­molecular O—H⋯O hydrogen bonds.

In (**I**), several π–π contacts are observed between inversion-related rings, with centroid–centroid distances ranging from 3.835 (3) to 3.966 (3) Å (Table 2 ▸). These inter­actions stack the mol­ecules into layered ribbons that extend along the *b*-axis direction. Compound (**II**) also exhibits aromatic stacking. *Cg*2⋯*Cg*2^i^ contacts [3.634 (5) Å; symmetry code: (i) 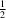 − *x*, 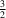 − *y*, 1 − *z*] and *Cg*1⋯*Cg*1^ii^ contacts [3.666 (4) Å; symmetry code: (ii) 1 − *x*, 2 − *y*, 1 − *z*] form columnar arrays running through the crystal. One set of columns runs along the [110] direction, and the other along the [1
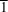
0] direction. Similarly, in (**III**), several strong π–π inter­actions are observed: *Cg*2⋯*Cg*2^i^ = 3.802 (3) Å [symmetry code: (i) 1 − *x*, −*y*, −*z*], *Cg*3⋯*Cg*3^ii^ = 3.632 (2) Å [symmetry code: (ii) 1 − *x*, 1 − *y*, 1 − *z*], and *Cg*4⋯*Cg*4^iii^ = 3.681 (2) Å [symmetry code: (iii) 1 − *x*, 2 − *y*, 1 − *z*]. These inter­actions result in columnar arrays running along the *b*-axis direction, with the columns connected by additional π–π inter­actions to form sheets in the *bc* plane.

In contrast, (**IV**) exhibits fewer and weaker contacts, with *Cg*2⋯*Cg*1^i^ at 3.9288 (13) Å [symmetry code: (i) 1 − *x*, 1 − *y*, 1 − *z*] being the only observed π–π stacking inter­action. Compound (**V**) has multiple π–π contacts, with centroid–centroid distances ranging from 3.7374 (19) to 3.969 (2) Å (Table 3 ▸). In addition to aromatic stacking, (**V**) is also consolidated by hydrogen bonds involving coordinated water mol­ecules and nitrate anions. Notable examples include O6—H6*A*⋯O9 [O⋯O = 2.723 (3) Å], O6—H6*B*⋯O2 [2.651 (3) Å], O5—H5*A*⋯O13^ii^ [2.861 (3) Å], O5—H5*B*⋯O9^iii^ [2.802 (3) Å], and O8—H8*B*⋯O12 [2.710 (3) Å]. These hydrogen bonds, with D—H⋯A angles often approaching linearity [*e.g.*, 177 (3)° for O6—H6*B*⋯O2], tie the complexes together into a robust three-dimensional network [symmetry codes: (ii) −*x* + 1, −*y* + 1, −*z* + 1; (iii) −*x* + 1, −*y*, –*z* + 1].

## 4. Hirshfeld surface analysis

**Figure 11:**
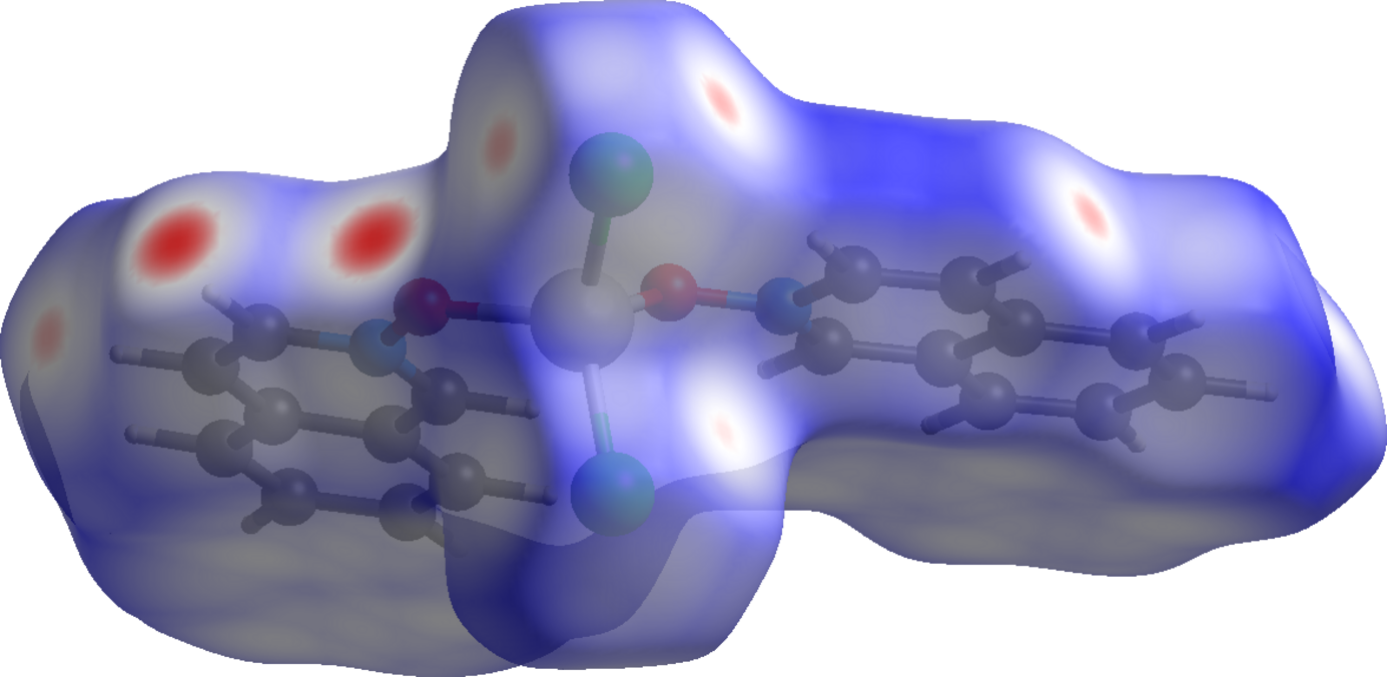
Hirshfeld surface for (**I**) mapped over *d*_norm_.

The inter­molecular inter­actions were further investigated by qu­anti­tative analysis of the Hirshfeld surfaces using *CrystalExplorer 21* (Spackman *et al.*, 2021 ▸), and visualized *via* two-dimensional fingerprint plots (McKinnon *et al.*, 2007 ▸). Figs. 11 ▸, 12 ▸ and 13 ▸ show the Hirshfeld surfaces of mol­ecules (**I**)–(**III**), each mapped with the function *d*_norm_, which is the sum of the distances from a surface point to the nearest inter­ior (*d*_i_) and exterior (*d*_e_) atoms, normalized by the van der Waals (vdW) radii of the corresponding atoms (*r*_vdW_). Contacts shorter than the sums of vdW radii are shown in red, those longer in blue, and those approximately equal to vdW in white.

For (**I**), (**II**), and (**III**), the most intense red spots correspond to C—H⋯*X* and C—H⋯O inter­actions. In (**I**), the short contact C10—H10⋯O2(2 − *x*, 1 − *y*, −*z*) has an H⋯O distance of 2.447 (3) Å. Additional short contacts include C11—H11⋯Cl2(2 − *x*, −*y*, −*z*) at 2.8305 (13) Å and C8—H8⋯Cl2(2 − *x*, 1 − *y*, 1 − *z*) at 2.8649 (13) Å. In (**II**), the most significant short contacts are C5—H5⋯Br1(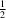 + *x*, 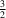 − *y*, −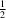 + *z*) at 2.9832 (6) Å and C4—H4⋯O1(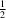 + *x*, −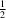 + *y*, *z*) at 2.670 (4) Å. In (**III**), the short contacts C4—H4⋯O1(*x*, 1 + *y*, *z*) at 2.669 (2) Å and C18—H18⋯O2(2 − *x*, 1 − *y*, 1 − *z*) at 2.566 (2) Å are also observed. All of these short contacts can be regarded as weak hydrogen bonds (Steiner, 1998 ▸).

**Figure 14:**
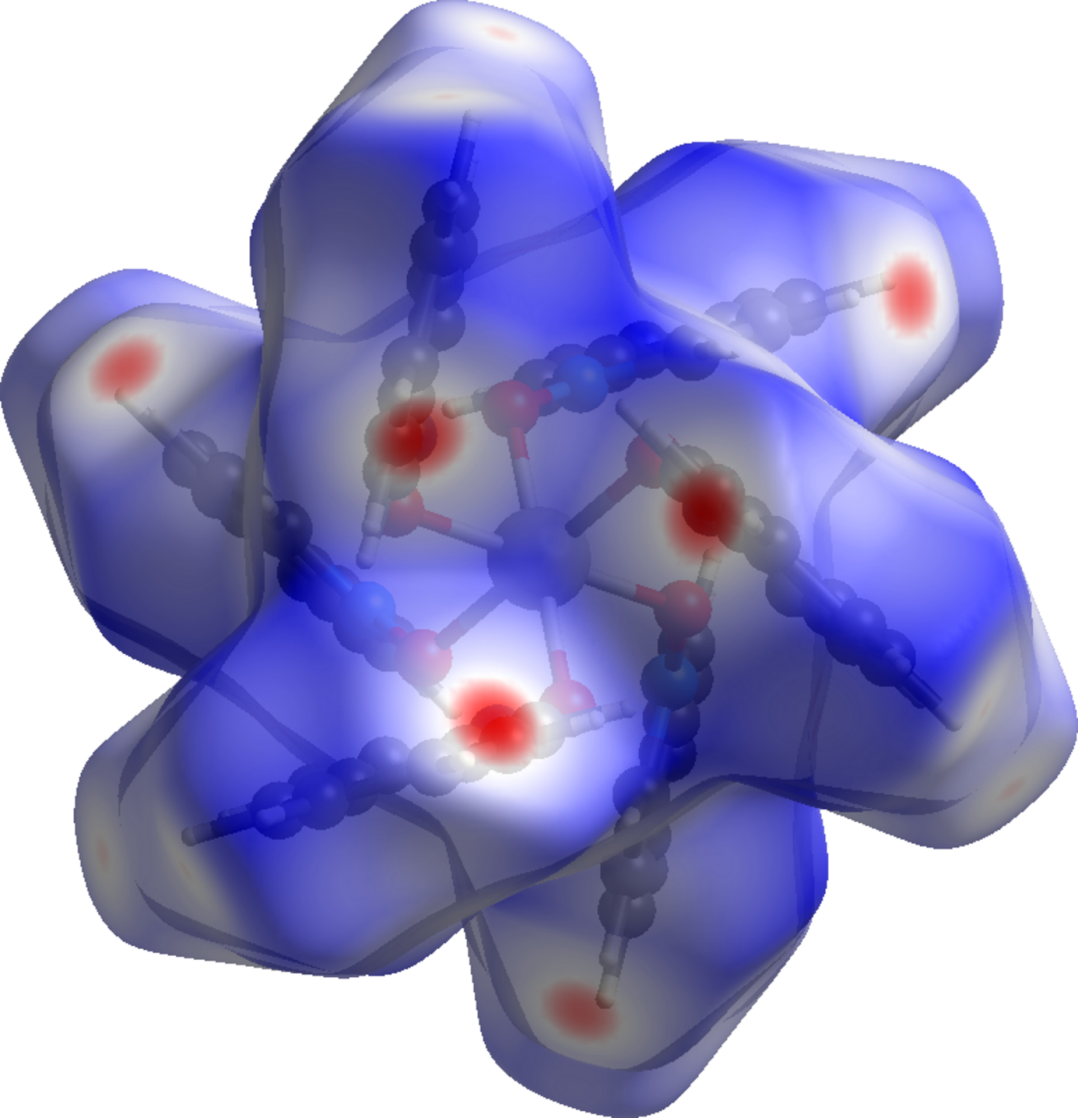
Hirshfeld surface for (**IV**) mapped over *d*_norm_.

**Figure 15:**
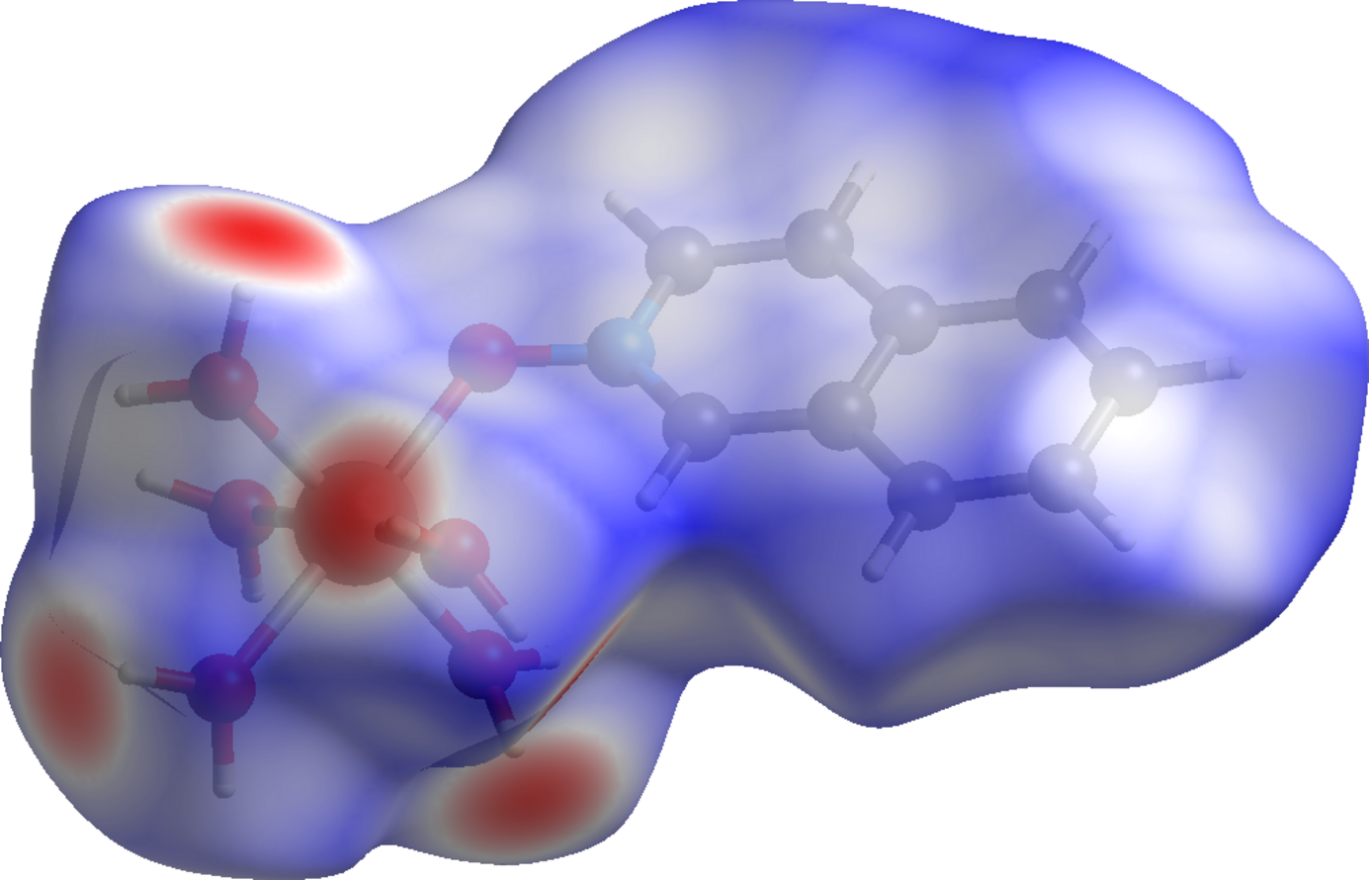
Hirshfeld surface for (**V**) mapped over *d*_norm_.

In (**IV**), (Fig. 14 ▸), the shortest contacts correspond to C—H⋯O inter­actions, primarily C4—H4⋯O3(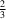 + *y* − *x*, 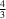 − *x*, 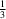 + *z*) at 2.4079 (18) Å. In (**V**), (Fig. 15 ▸), the closest contacts are the hydrogen bonds between IQNO and the water mol­ecules, and between the nitrate ions and water described above; there are also weak hydrogen bonds involving C1—H1⋯O2(−*x*, 1 − *y*, 1 − *z*) at 2.319 (2) Å and C19—H19⋯O1(−*x*, 1 − *y*, 1 − *z*) at 2.402 (2) Å.

Analysis of the two-dimensional fingerprint plots (Table 4 ▸) indicates that H⋯H contacts are the most common in all five structures. In (**I**)–(**III**), the *X*⋯H contacts constitute the second-highest contribution, which increases in the order (**I**) < (**II**) < (**III**), contributing 29.0%, 30.9%, and 31.1%, respectively. In (**I**V) and (**V**), the Hirshfeld surface for the Zn complex was used in the analysis, and O⋯H contacts form the second-highest contribution, contributing 24.5%, 37.6%, and 31.1%, respectively. No short halogen⋯halogen contacts are observed in (**I**)–(**III**).

## 5. Database survey

A search of the Cambridge Structural Database (CSD, version 5.42, update September 2022; Groom *et al.*, 2016 ▸) for iso­quinoline *N*-oxide returned 14 unique entries. Of these 14, only 5 were bound directly to metal atoms. The most closely related to these complexes are cobalt(II) [CSD refcodes PINNUX (Munn *et al.*, 2014 ▸) and QIWWEB (Kawamura *et al.*, 2019 ▸)], niobium(III) (QARFAU; Sperlich & Kockerling, 2022 ▸), zinc(II) (UWIPAS; Oberda *et al.*, 2011 ▸), and osmium(VIII) (XONBIP; Calabrese *et al.*, 2024 ▸).

When the seven hydrogen atoms are removed in the substructure search, the number of unique entries increases to 72, with four additional metal-bound examples not mentioned above. These include a 1-sulfanyl-iso­quinoline ruthenium(II) complex (MUTSAY; Kladnik *et al.*, 2020 ▸), a 1-(­oxy)-3-iso­quinoline-*N*-oxide-carboxamidato derivative with indium(III) (VOLNIU; Seitz *et al.*, 2008 ▸), a sodium derivative of iQNO with amino/crown ether attachment (ZEXCAG; Suwińska, 1995 ▸), and a europium(II) iQNO derivative modified with a cyclic bipyridyl (ZODXIZ; Paul-Roth *et al.*, 1995 ▸).

## 6. Synthesis and crystallization

The title compounds were all synthesized in a similar manner. The zinc salt was dissolved in ∼10 ml of methanol, and then iso­quinoline *N*-oxide (iQNO) was added in one portion. The solutions were stirred for 5 minutes, and the solvent was allowed to evaporate, resulting in crystalline solids over time.

Compound (**I**) was prepared by adding ZnCl_2_ (0.0463 g, 0.340 mmol, purchased from Strem Chemicals) to a small portion of methanol to dissolve, and adding 0.100 g of iQNO (0.689 mmol, purchased from Aldrich/Millipore) in a 1:2 zinc(II):iQNO mole ratio. The solution was stirred for approximately 10 minutes, at which time the solution was covered with parafilm, and the solvent was allowed to evaporate at 295 K. Yield: 0.123 g (84.1%).

Compound (**II**) was synthesized by placing 0.0818 g (0.340 mmol, purchased from Alfa Aesar) of ZnBr_2_·0.86H_2_O in a small beaker and dissolving it in minimal amounts of methanol. iQNO (0.100 g, 0.689 mmol, purchased from Aldrich/Millipore) was added in one portion. The mixture was stirred for 10 minutes, covered with parafilm, and allowed to evaporate at 295 K. Yield: 0.138 g (75.9%).

For compound (**III**), a similar technique was used. ZnI_2_ (0.109 g, 0.340 mmol, purchased from Aldrich/Millipore) was placed in a beaker, and methanol was added to dissolve it completely. iQNO (0.100 g, 0.689 mmol, purchased from Aldrich/Millipore) was added in one portion. The mixture was stirred for 10 minutes, covered with parafilm, and allowed to evaporate at 295 K. Yield: 0.146 g (69.8%).

Complex (**IV**) was prepared in a 1:4 zinc(II):iQNO ratio by dissolving 0.0633 g (0.0170 mmol, purchased from Aldrich/Millipore) of Zn(ClO_4_)_2_·6H_2_O in methanol and adding 0.100 g (0.689 mmol) of iQNO in one portion. The solution was stirred for 10 minutes, and the solvent was evaporated to a minimum amount of liquid. This liquid was redissolved in tetra­hydro­furan, dried over MgSO_4_, and the solvent was evaporated. The resulting solid was dissolved in aceto­nitrile, which was allowed to evaporate at room temperature, yielding the product. Yield: 0.0423 g (32.4% based on iQNO).

Compound (**V**) was synthesized by dissolving 0.0994 g (0.340 mmol, purchased from Alfa Aesar) of Zn(NO_3_)_2_^.^6H_2_O in methanol and adding 0.100 g of iQNO (0.689 mmol) in a 1:2 ratio. The same procedure as outlined for (**IV**) was followed, with a final yield of 0.0642 g (39.1% based on iQNO).

## 7. Refinement

Crystal data, data collection and structure refinement details are summarized in Table 5 ▸. All carbon-bound H atoms were positioned geometrically and refined as riding: C—H = 0.95–0.98 Å with *U*_iso_(H) = 1.2*U*_eq_(C).

## Supplementary Material

Crystal structure: contains datablock(s) I, II, III, IV, V. DOI: 10.1107/S2056989025000180/hb8119sup1.cif

Structure factors: contains datablock(s) I. DOI: 10.1107/S2056989025000180/hb8119Isup2.hkl

Structure factors: contains datablock(s) II. DOI: 10.1107/S2056989025000180/hb8119IIsup3.hkl

Structure factors: contains datablock(s) III. DOI: 10.1107/S2056989025000180/hb8119IIIsup4.hkl

Structure factors: contains datablock(s) IV. DOI: 10.1107/S2056989025000180/hb8119IVsup5.hkl

Structure factors: contains datablock(s) V. DOI: 10.1107/S2056989025000180/hb8119Vsup6.hkl

CCDC references: 2415566, 2415565, 2415564, 2415563, 2415562

Additional supporting information:  crystallographic information; 3D view; checkCIF report

## Figures and Tables

**Figure 7:**
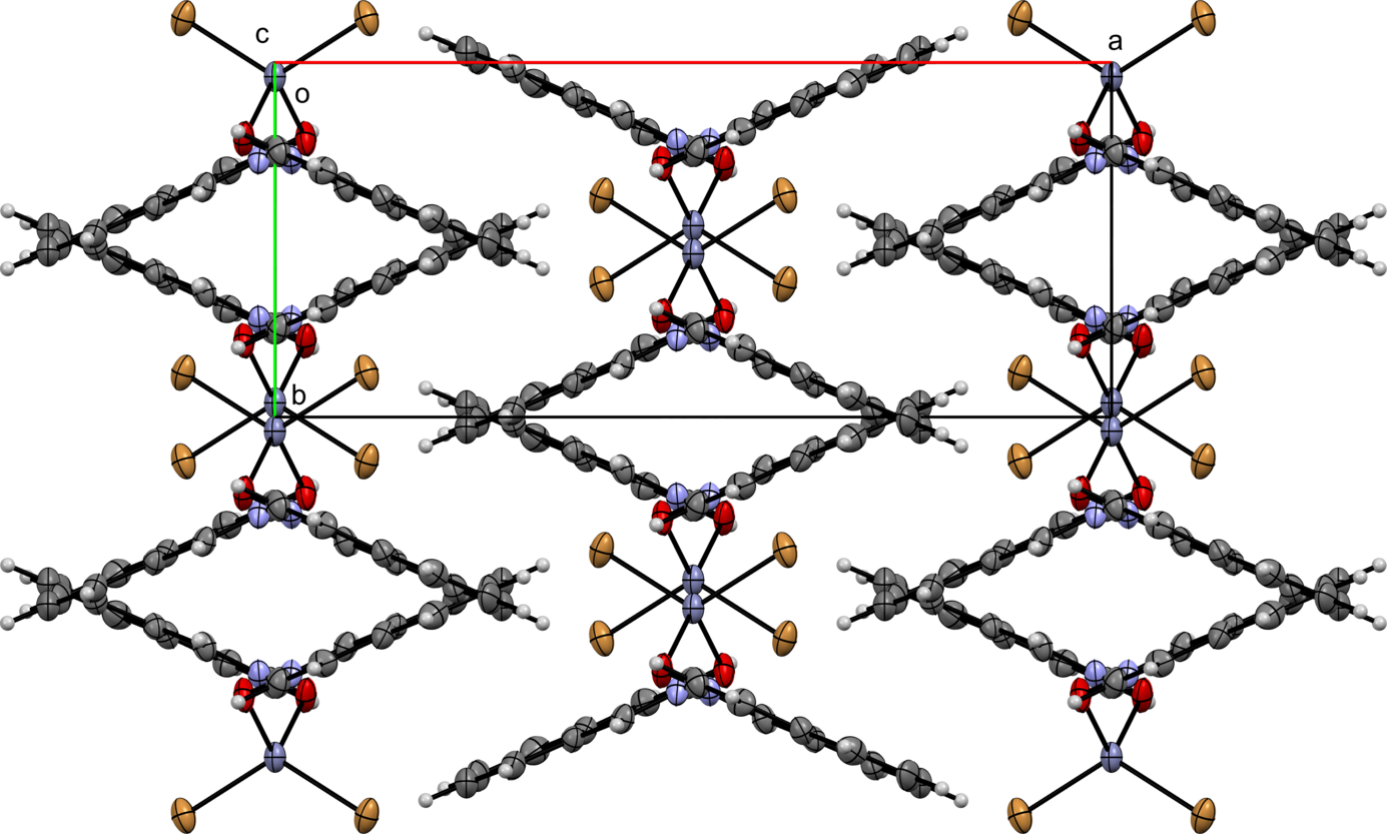
A view along the *c-*axis direction of the crystal packing of (**II**).

**Figure 8:**
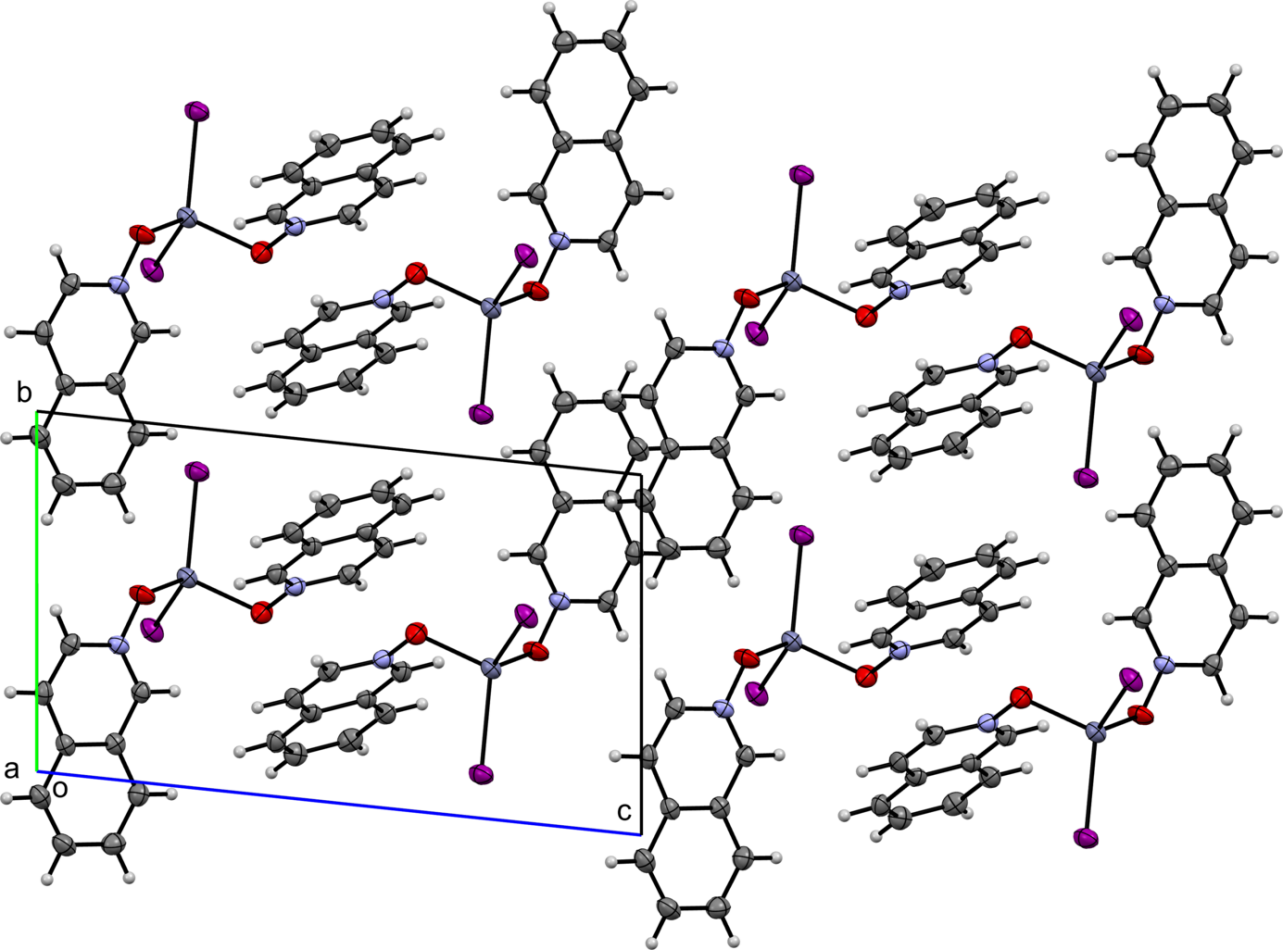
A view along the *a-*axis direction of the crystal packing of (**III**).

**Figure 9:**
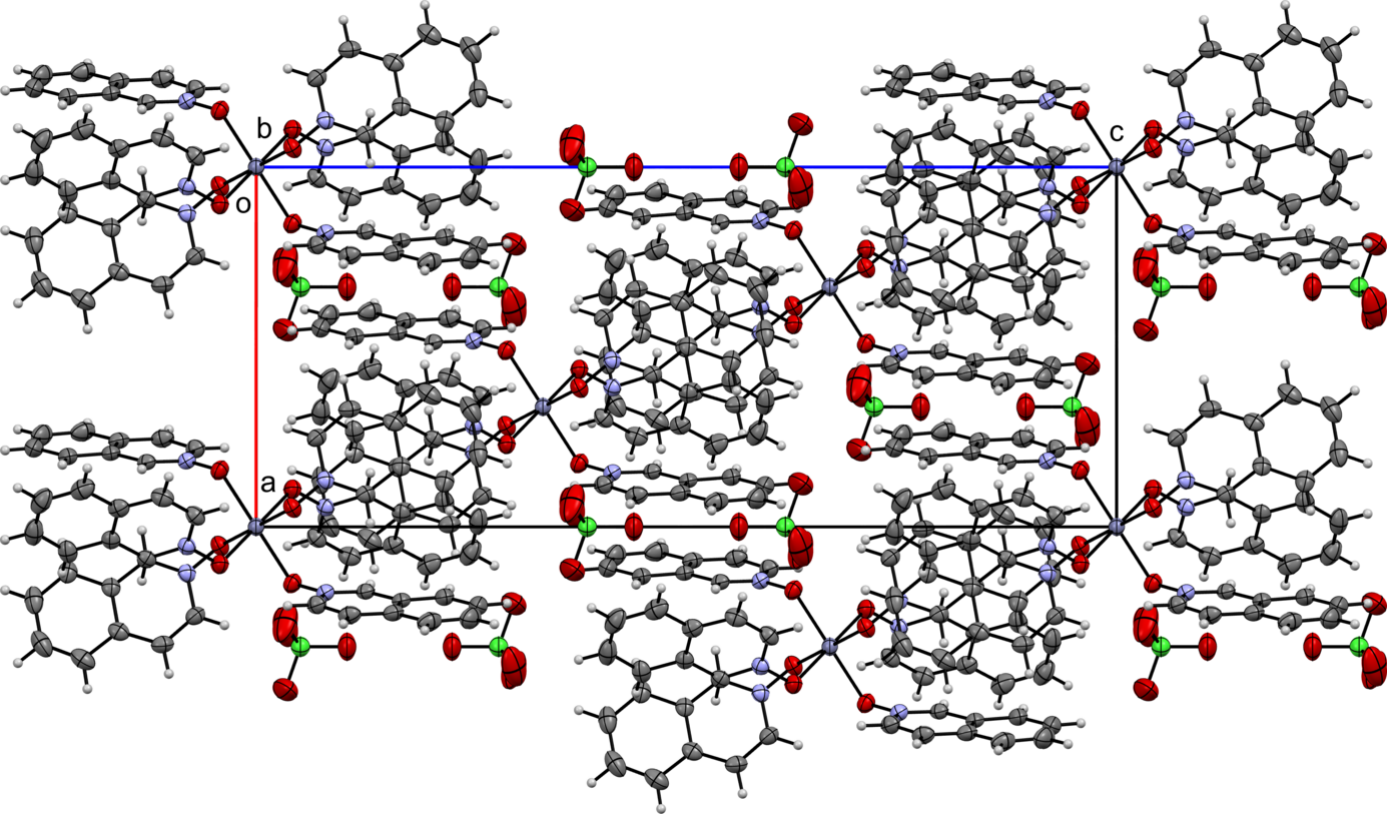
A view along the *b-*axis direction of the crystal packing of (**IV**).

**Figure 10:**
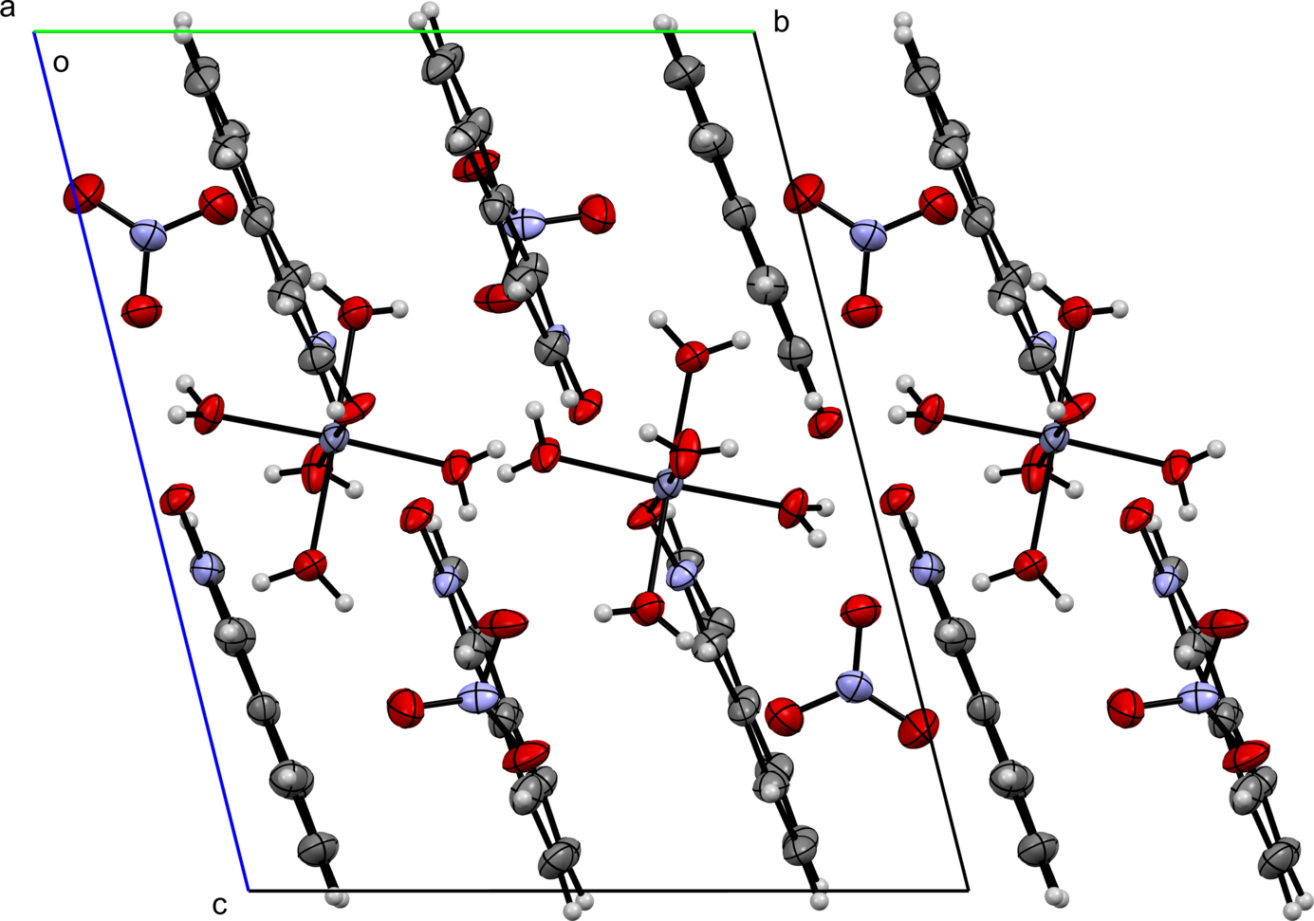
A view along the *a-*axis direction of the crystal packing of (**V**).

**Figure 12:**
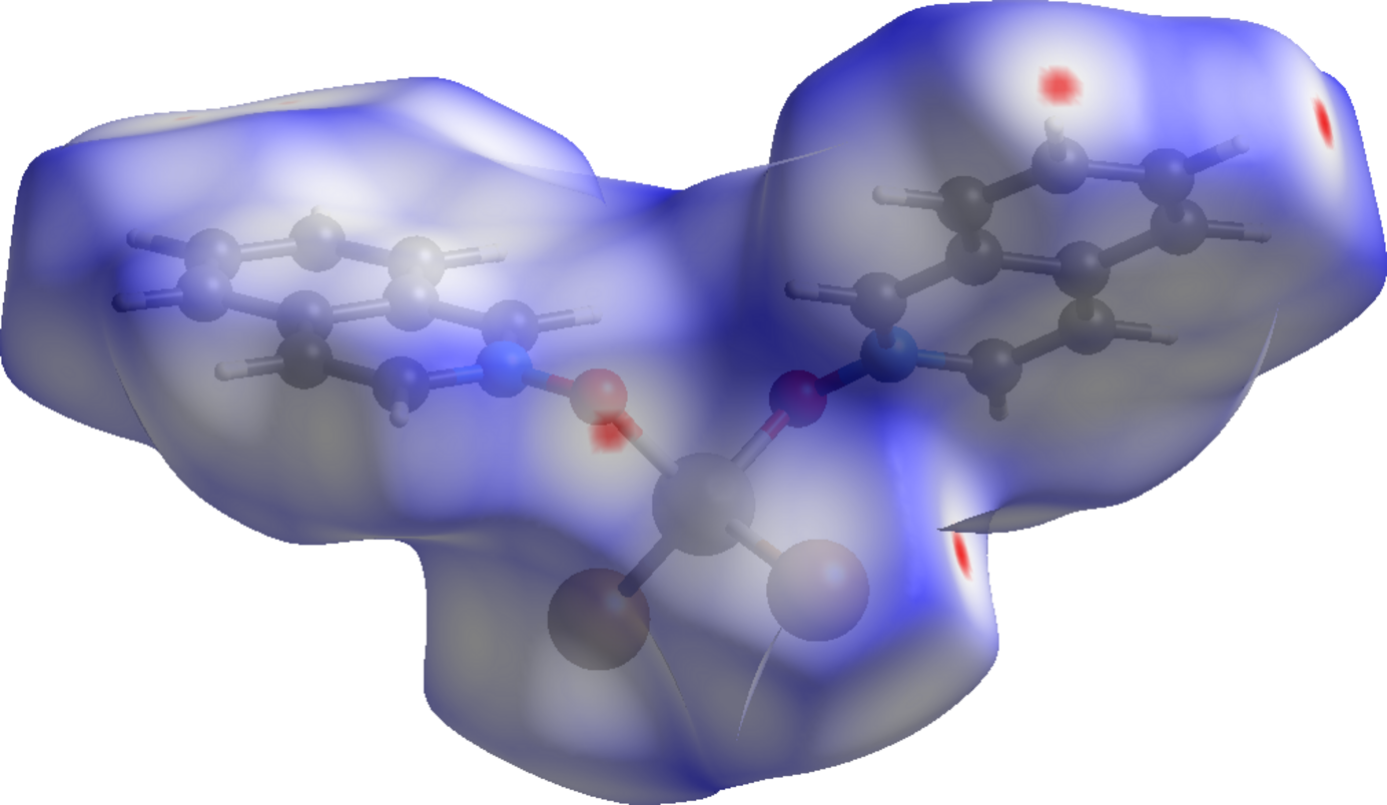
Hirshfeld surface for (**II**) mapped over *d*_norm_.

**Figure 13:**
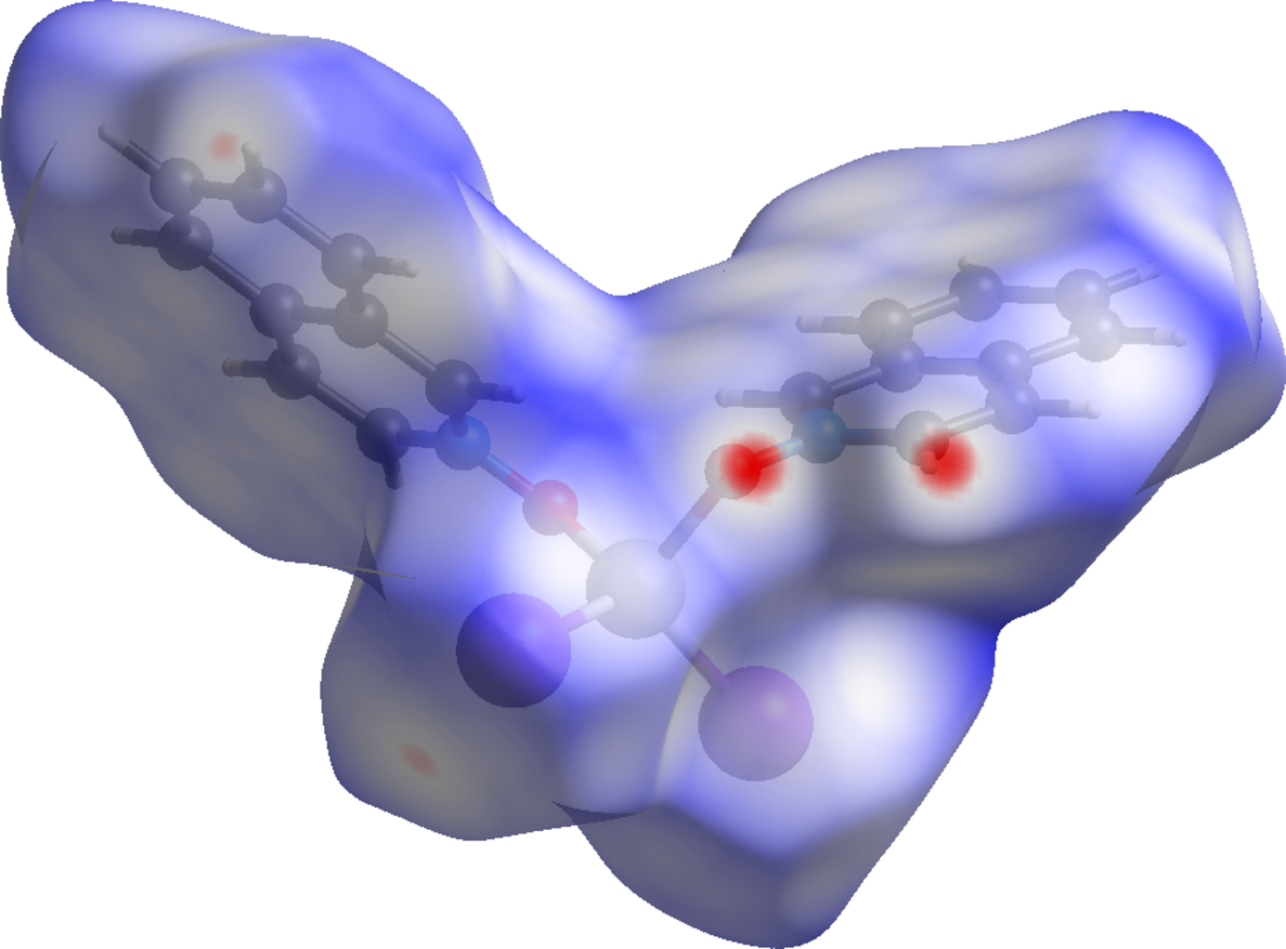
Hirshfeld surface for (**III**) mapped over *d*_norm_.

**Table 1:** Hydrogen-bond geometry (Å, °) for (**V**)

*D*—H⋯*A*	*D*—H	H⋯*A*	*D*⋯*A*	*D*—H⋯*A*
O6—H6*A*⋯O9	0.82 (2)	1.91 (2)	2.723 (3)	173 (4)
O5—H5*A*⋯O13^ii^	0.83 (2)	2.04 (2)	2.861 (3)	168 (3)
O8—H8*A*⋯O10	0.83 (2)	2.00 (2)	2.808 (3)	164 (4)
O5—H5*B*⋯O9^iii^	0.83 (2)	1.98 (2)	2.802 (3)	171 (4)
O6—H6*B*⋯O2	0.82 (2)	1.84 (2)	2.651 (3)	177 (3)
O7—H7*A*⋯O3^ii^	0.82 (2)	2.03 (2)	2.798 (3)	156 (3)
O8—H8*B*⋯O12	0.83 (2)	1.88 (2)	2.710 (3)	179 (4)
O7—H7*B*⋯O2^iii^	0.82 (2)	1.94 (2)	2.755 (3)	173 (4)
O4—H4*A*⋯O3	0.82 (2)	1.81 (2)	2.634 (3)	176 (5)
O4—H4*B*⋯O12^ii^	0.81 (2)	1.93 (2)	2.729 (3)	169 (4)

Symmetry codes: (ii) 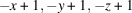; (iii) 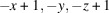.

**Table 2:** Centroid distances (Å) for (**I**) *Cg*–*Cg*4 are the centroids of the N1/C1/C2/C7–C9, N2/C10–C12/C17/C18, C2–C7 and C12–C17 rings, respectively.

*Cg*1⋯*Cg*1^i^	3.928 (4)	*Cg*3⋯*Cg*3^ii^	3.845 (4)
*Cg*1⋯*Cg*3^i^	3.966 (3)	*Cg*4⋯*Cg*4^iv^	3.681 (4)
*Cg*1⋯*Cg*3^ii^	3.835 (3)	*Cg*4⋯*Cg*2^iii^	3.940 (3)
*Cg*2⋯*Cg*2^iii^	3.437 (3)	*Cg*4⋯*Cg*2^iv^	3.906 (3)

Symmetry codes: (i) 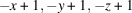; (ii) 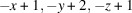; (iii) 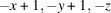; (iv) 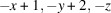.

**Table 3:** Centroid distances (Å) for (**V**) *Cg*1, *Cg*2, *Cg*4, *Cg*5, *Cg*7 and *Cg*8 are the centroids of the N1/C1–C3/C8/C9, C3–C8, N2/C10–C12/C17/C18, C12–C17, N3/C19–C21/C26/C27 and C21–C26 rings, respectively.

*Cg*1⋯*Cg*4	3.839 (2)	*Cg*2⋯*Cg*8	3.969 (2)
*Cg*1⋯*Cg*7	3.893 (2)	*Cg*4⋯*Cg*7^i^	3.943 (2)
*Cg*1⋯*Cg*8	3.8500 (19)	*Cg*5⋯*Cg*7^i^	3.7374 (19)
*Cg*2⋯*Cg*4	3.6617 (19)	*Cg*5⋯*Cg*8^i^	3.968 (2)
*Cg*2⋯*Cg*5	3.823 (2)		

Symmetry code: (i) 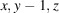.

**Table 4:** Contributions of selected inter­molecular contacts (%) to the Hirshfeld surfaces of (**I**)–(**V**)

Compound	(**I**)	(**II**)	(**III**)	(**IV**)	(**V**)
H⋯H	30.9	27.8	28.0	41.8	40.6
H⋯*X*/*X*⋯H	29.0	30.9	31.1	–	–
C⋯H/H⋯C	13.3	20.1	17.2	22.0	8.6
C⋯C	11.3	6.6	7.0	6.0	8.5
O⋯H/H⋯O	8.8	8.3	7.9	24.5	37.6

**Table 5:** Experimental details

	(**I**)	(**II**)	(**III**)	(**IV**)	(**V**)
Crystal data					
Chemical formula	[ZnCl_2_(C_9_H_7_NO)_2_]	[ZnBr_2_(C_9_H_7_NO)_2_]	[ZnI_2_(C_9_H_7_NO)_2_]	[Zn(C_9_H_7_NO)_6_](ClO_4_)_2_	[Zn(C_9_H_7_NO)(H_2_O)_5_](NO_3_)_2_·2C_9_H_7_NO
*M*_r_	426.58	515.50	609.48	1135.20	714.94
Crystal system, space group	Triclinic, *P* 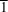	Monoclinic, *C*2/*c*	Triclinic, *P* 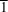	Trigonal, *R* 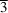	Triclinic, *P* 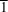
Temperature (K)	293	170	170	170	170
*a*, *b*, *c* (Å)	7.5164 (5), 7.8002 (5), 15.1156 (10)	17.1095 (10), 7.2020 (6), 14.9534 (11)	7.7325 (3), 8.9596 (4), 14.8297 (9)	12.8217 (4), 12.8217 (4), 26.5684 (9)	9.7360 (9), 11.5910 (9), 14.6381 (10)
α, β, γ (°)	96.172 (6), 92.052 (5), 96.284 (5)	90, 96.472 (6), 90	93.205 (4), 99.873 (4), 104.852 (4)	90, 90, 120	74.352 (6), 74.620 (7), 81.633 (7)
*V* (Å^3^)	874.77 (10)	1830.9 (2)	972.98 (9)	3782.6 (3)	1528.8 (2)
*Z*	2	4	2	3	2
Radiation type	Mo *K*α	Mo *K*α	Mo *K*α	Mo *K*α	Mo *K*α
μ (mm^−1^)	1.72	5.72	4.45	0.67	0.88
Crystal size (mm)	0.4 × 0.3 × 0.1	0.2 × 0.2 × 0.2	0.32 × 0.13 × 0.06	0.2 × 0.2 × 0.15	0.7 × 0.2 × 0.04

Data collection					
Diffractometer	XtaLAB Synergy, HyPix3000	XtaLAB Mini	XtaLAB Mini	XtaLAB Mini	XtaLAB Mini
Absorption correction	Multi-scan (*CrysAlis PRO*; Rigaku OD, 2019 ▸)	Multi-scan (*CrysAlis PRO*; Rigaku OD, 2019 ▸)	Multi-scan (*CrysAlis PRO*; Rigaku OD, 2019 ▸)	Multi-scan (*CrysAlis PRO*; Rigaku OD, 2019 ▸)	Multi-scan (*CrysAlis PRO*; Rigaku OD, 2019 ▸)
*T*_min_, *T*_max_	0.929, 1.000	0.358, 1.000	0.535, 1.000	0.914, 1.000	0.752, 1.000
No. of measured, independent and observed [*I* > 2σ(*I*)] reflections	4838, 3207, 2357	2980, 1671, 1402	8495, 3563, 3152	11238, 1545, 1450	13628, 5599, 4252
*R*_int_	0.029	0.044	0.016	0.017	0.047
(sin θ/λ)_max_ (Å^−1^)	0.602	0.602	0.602	0.602	0.602

Refinement					
*R*[*F*^2^ > 2σ(*F*^2^)], *wR*(*F*^2^), *S*	0.049, 0.124, 1.06	0.052, 0.141, 1.03	0.020, 0.048, 1.03	0.028, 0.077, 1.06	0.044, 0.106, 1.04
No. of reflections	3207	1671	3563	1545	5599
No. of parameters	226	114	226	117	464
No. of restraints	0	0	0	0	10
H-atom treatment	H-atom parameters constrained	H-atom parameters constrained	H-atom parameters constrained	H-atom parameters constrained	H atoms treated by a mixture of independent and constrained refinement
Δρ_max_, Δρ_min_ (e Å^−3^)	0.55, −0.30	0.83, −0.96	0.59, −0.39	0.30, −0.25	0.51, −0.48

Computer programs: *CrysAlis PRO* (Rigaku OD, 2019 ▸), *SHELXT* (Sheldrick, 2015*a* ▸), *SHELXL* (Sheldrick, 2015*b* ▸) and *OLEX2* (Dolomanov *et al.*, 2009 ▸).

## supplementary crystallographic information

### Dichloridobis(isoquinoline N-oxide-κO)zinc(II) (I) Crystal data

**Table:**

[ZnCl_2_(C_9_H_7_NO)_2_]	*Z* = 2
*M**_r_* = 426.58	*F*(000) = 432
Triclinic, *P*1	*D*_x_ = 1.620 Mg m^−^^3^
*a* = 7.5164 (5) Å	Mo *K*α radiation, λ = 0.71073 Å
*b* = 7.8002 (5) Å	Cell parameters from 520 reflections
*c* = 15.1156 (10) Å	θ = 2.7–22.6°
α = 96.172 (6)°	µ = 1.72 mm^−^^1^
β = 92.052 (5)°	*T* = 293 K
γ = 96.284 (5)°	Irregular, clear colourless
*V* = 874.77 (10) Å^3^	0.4 × 0.3 × 0.1 mm

### Dichloridobis(isoquinoline N-oxide-κO)zinc(II) (I) Data collection

**Table:**

XtaLAB Synergy, HyPix3000 diffractometer	3207 independent reflections
Radiation source: fine-focus sealed X-ray tube, Rigaku (Mo) X-ray Source	2357 reflections with *I* > 2σ(*I*)
Graphite monochromator	*R*_int_ = 0.029
ω scans	θ_max_ = 25.4°, θ_min_ = 2.6°
Absorption correction: multi-scan (CrysAlisPro; Rigaku OD, 2019)	*h* = −9→9
*T*_min_ = 0.929, *T*_max_ = 1.000	*k* = −9→8
4838 measured reflections	*l* = −18→18

### Dichloridobis(isoquinoline N-oxide-κO)zinc(II) (I) Refinement

**Table:**

Refinement on *F*^2^	Primary atom site location: dual
Least-squares matrix: full	Hydrogen site location: inferred from neighbouring sites
*R*[*F*^2^ > 2σ(*F*^2^)] = 0.049	H-atom parameters constrained
*wR*(*F*^2^) = 0.124	*w* = 1/[σ^2^(*F*_o_^2^) + (0.0519*P*)^2^ + 0.0439*P*] where *P* = (*F*_o_^2^ + 2*F*_c_^2^)/3
*S* = 1.06	(Δ/σ)_max_ < 0.001
3207 reflections	Δρ_max_ = 0.55 e Å^−^^3^
226 parameters	Δρ_min_ = −0.30 e Å^−^^3^
0 restraints	

### Dichloridobis(isoquinoline N-oxide-κO)zinc(II) (I) Special details

**Table:**

Geometry. All esds (except the esd in the dihedral angle between two l.s. planes) are estimated using the full covariance matrix. The cell esds are taken into account individually in the estimation of esds in distances, angles and torsion angles; correlations between esds in cell parameters are only used when they are defined by crystal symmetry. An approximate (isotropic) treatment of cell esds is used for estimating esds involving l.s. planes.

### Dichloridobis(isoquinoline N-oxide-κO)zinc(II) (I) Fractional atomic coordinates and isotropic or equivalent isotropic displacement parameters (Å^2^)

**Table:**

	*x*	*y*	*z*	*U*_iso_*/*U*_eq_	
Zn1	0.94135 (7)	0.67381 (7)	0.23426 (3)	0.0471 (2)	
Cl2	1.10215 (18)	0.47387 (17)	0.27790 (8)	0.0642 (4)	
Cl1	1.0537 (2)	0.94814 (17)	0.26629 (9)	0.0698 (4)	
O2	0.8996 (4)	0.6204 (4)	0.10445 (19)	0.0526 (8)	
O1	0.6934 (4)	0.6319 (5)	0.27690 (19)	0.0628 (9)	
N2	0.7659 (5)	0.6628 (4)	0.0538 (2)	0.0433 (9)	
N1	0.6497 (5)	0.6711 (5)	0.3619 (2)	0.0465 (9)	
C1	0.4937 (6)	0.7275 (6)	0.3769 (3)	0.0474 (11)	
H1	0.417826	0.742590	0.329117	0.057*	
C16	0.3419 (7)	0.8481 (6)	0.0684 (3)	0.0558 (12)	
H16	0.340539	0.880666	0.129381	0.067*	
C10	0.7735 (6)	0.6177 (6)	−0.0366 (3)	0.0473 (11)	
H10	0.870860	0.566543	−0.059286	0.057*	
C2	0.4403 (6)	0.7651 (5)	0.4644 (3)	0.0444 (10)	
C7	0.5573 (6)	0.7419 (5)	0.5355 (3)	0.0450 (11)	
C11	0.6378 (7)	0.6485 (6)	−0.0920 (3)	0.0543 (12)	
H11	0.643301	0.618191	−0.152943	0.065*	
C18	0.6327 (6)	0.7382 (6)	0.0875 (3)	0.0464 (11)	
H18	0.633820	0.769454	0.148655	0.056*	
C6	0.5010 (8)	0.7816 (7)	0.6236 (3)	0.0622 (14)	
H6	0.575863	0.767927	0.672033	0.075*	
C8	0.7216 (6)	0.6805 (6)	0.5152 (3)	0.0528 (12)	
H8	0.801520	0.663881	0.561100	0.063*	
C3	0.2723 (6)	0.8255 (6)	0.4807 (3)	0.0541 (12)	
H3	0.195615	0.841291	0.433315	0.065*	
C17	0.4855 (6)	0.7738 (5)	0.0328 (3)	0.0445 (11)	
C9	0.7644 (6)	0.6456 (6)	0.4302 (3)	0.0544 (12)	
H9	0.873034	0.603814	0.417646	0.065*	
C14	0.2034 (7)	0.8278 (7)	−0.0791 (4)	0.0684 (15)	
H14	0.108847	0.849968	−0.115940	0.082*	
C12	0.4906 (6)	0.7244 (5)	−0.0598 (3)	0.0467 (11)	
C15	0.2012 (7)	0.8732 (7)	0.0129 (4)	0.0640 (14)	
H15	0.102906	0.921058	0.036766	0.077*	
C5	0.3395 (8)	0.8389 (7)	0.6375 (3)	0.0678 (15)	
H5	0.304849	0.864656	0.695408	0.081*	
C4	0.2242 (7)	0.8599 (7)	0.5655 (3)	0.0626 (14)	
H4	0.113135	0.898061	0.576242	0.075*	
C13	0.3403 (7)	0.7526 (7)	−0.1148 (3)	0.0615 (13)	
H13	0.337385	0.718600	−0.175771	0.074*	

### Dichloridobis(isoquinoline N-oxide-κO)zinc(II) (I) Atomic displacement parameters (Å^2^)

**Table:**

	*U*^11^	*U*^22^	*U*^33^	*U*^12^	*U*^13^	*U*^23^
Zn1	0.0432 (3)	0.0575 (4)	0.0421 (3)	0.0109 (2)	0.0037 (2)	0.0062 (2)
Cl2	0.0699 (9)	0.0717 (8)	0.0564 (7)	0.0294 (7)	−0.0025 (6)	0.0127 (6)
Cl1	0.0879 (11)	0.0572 (8)	0.0628 (8)	0.0093 (7)	0.0064 (7)	−0.0027 (6)
O2	0.045 (2)	0.070 (2)	0.0453 (17)	0.0194 (16)	−0.0029 (15)	0.0053 (15)
O1	0.049 (2)	0.101 (3)	0.0377 (17)	0.0051 (18)	0.0113 (15)	0.0018 (17)
N2	0.040 (2)	0.046 (2)	0.044 (2)	0.0067 (17)	0.0032 (17)	0.0074 (17)
N1	0.037 (2)	0.064 (2)	0.039 (2)	0.0006 (17)	0.0053 (17)	0.0110 (18)
C1	0.039 (3)	0.058 (3)	0.045 (2)	0.001 (2)	−0.002 (2)	0.012 (2)
C16	0.051 (3)	0.058 (3)	0.063 (3)	0.011 (2)	0.013 (3)	0.016 (2)
C10	0.051 (3)	0.051 (3)	0.040 (2)	0.009 (2)	0.004 (2)	0.005 (2)
C2	0.040 (3)	0.047 (3)	0.046 (2)	0.003 (2)	0.003 (2)	0.007 (2)
C7	0.046 (3)	0.048 (3)	0.042 (2)	0.001 (2)	0.002 (2)	0.013 (2)
C11	0.062 (3)	0.060 (3)	0.041 (2)	0.008 (2)	0.005 (2)	0.002 (2)
C18	0.051 (3)	0.049 (3)	0.041 (2)	0.008 (2)	0.002 (2)	0.008 (2)
C6	0.074 (4)	0.075 (4)	0.039 (3)	0.011 (3)	0.003 (3)	0.013 (2)
C8	0.043 (3)	0.070 (3)	0.048 (3)	0.007 (2)	−0.004 (2)	0.019 (2)
C3	0.044 (3)	0.065 (3)	0.054 (3)	0.008 (2)	0.002 (2)	0.006 (2)
C17	0.043 (3)	0.045 (3)	0.047 (3)	0.001 (2)	0.008 (2)	0.011 (2)
C9	0.042 (3)	0.073 (3)	0.051 (3)	0.012 (2)	0.006 (2)	0.014 (2)
C14	0.052 (3)	0.069 (4)	0.088 (4)	0.006 (3)	−0.011 (3)	0.030 (3)
C12	0.047 (3)	0.037 (2)	0.056 (3)	0.0006 (19)	0.003 (2)	0.009 (2)
C15	0.042 (3)	0.066 (3)	0.090 (4)	0.012 (2)	0.006 (3)	0.031 (3)
C5	0.080 (4)	0.076 (4)	0.050 (3)	0.013 (3)	0.022 (3)	0.009 (3)
C4	0.058 (3)	0.068 (3)	0.062 (3)	0.013 (3)	0.012 (3)	0.002 (3)
C13	0.064 (4)	0.063 (3)	0.056 (3)	0.003 (3)	−0.011 (3)	0.014 (2)

### Dichloridobis(isoquinoline N-oxide-κO)zinc(II) (I) Geometric parameters (Å, º)

**Table:**

Zn1—Cl2	2.2147 (13)	C11—H11	0.9300
Zn1—Cl1	2.2088 (14)	C11—C12	1.390 (6)
Zn1—O2	1.968 (3)	C18—H18	0.9300
Zn1—O1	1.999 (3)	C18—C17	1.426 (6)
O2—N2	1.332 (4)	C6—H6	0.9300
O1—N1	1.349 (4)	C6—C5	1.354 (7)
N2—C10	1.378 (5)	C8—H8	0.9300
N2—C18	1.308 (5)	C8—C9	1.343 (6)
N1—C1	1.316 (5)	C3—H3	0.9300
N1—C9	1.366 (6)	C3—C4	1.353 (6)
C1—H1	0.9300	C17—C12	1.414 (6)
C1—C2	1.405 (6)	C9—H9	0.9300
C16—H16	0.9300	C14—H14	0.9300
C16—C17	1.379 (6)	C14—C15	1.400 (7)
C16—C15	1.370 (7)	C14—C13	1.340 (7)
C10—H10	0.9300	C12—C13	1.429 (7)
C10—C11	1.355 (6)	C15—H15	0.9300
C2—C7	1.404 (6)	C5—H5	0.9300
C2—C3	1.416 (6)	C5—C4	1.401 (7)
C7—C6	1.424 (6)	C4—H4	0.9300
C7—C8	1.405 (6)	C13—H13	0.9300
			
*Cg*1···*Cg*1^i^	3.928 (4)	*Cg*3···*Cg*3^ii^	3.845 (4)
*Cg*1···*Cg*3^i^	3.966 (3)	*Cg*4···*Cg*4^iv^	3.681 (4)
*Cg*1···*Cg*3^ii^	3.835 (3)	*Cg*4···*Cg*2^iii^	3.940 (3)
*Cg*2···*Cg*2^iii^	3.437 (3)	*Cg*4···*Cg*2^iv^	3.906 (3)
			
Cl1—Zn1—Cl2	117.35 (6)	C17—C18—H18	119.1
O2—Zn1—Cl2	106.39 (9)	C7—C6—H6	119.7
O2—Zn1—Cl1	109.57 (10)	C5—C6—C7	120.7 (5)
O2—Zn1—O1	101.78 (13)	C5—C6—H6	119.7
O1—Zn1—Cl2	109.07 (12)	C7—C8—H8	119.7
O1—Zn1—Cl1	111.43 (11)	C9—C8—C7	120.6 (4)
N2—O2—Zn1	127.6 (2)	C9—C8—H8	119.7
N1—O1—Zn1	124.0 (3)	C2—C3—H3	120.2
O2—N2—C10	115.9 (3)	C4—C3—C2	119.6 (5)
C18—N2—O2	122.4 (4)	C4—C3—H3	120.2
C18—N2—C10	121.6 (4)	C16—C17—C18	121.8 (4)
O1—N1—C9	119.7 (4)	C16—C17—C12	121.2 (4)
C1—N1—O1	118.8 (4)	C12—C17—C18	116.9 (4)
C1—N1—C9	121.5 (4)	N1—C9—H9	119.7
N1—C1—H1	119.6	C8—C9—N1	120.5 (4)
N1—C1—C2	120.8 (4)	C8—C9—H9	119.7
C2—C1—H1	119.6	C15—C14—H14	119.6
C17—C16—H16	120.4	C13—C14—H14	119.6
C15—C16—H16	120.4	C13—C14—C15	120.8 (5)
C15—C16—C17	119.3 (5)	C11—C12—C17	118.8 (4)
N2—C10—H10	120.3	C11—C12—C13	123.8 (4)
C11—C10—N2	119.4 (4)	C17—C12—C13	117.4 (4)
C11—C10—H10	120.3	C16—C15—C14	120.8 (5)
C1—C2—C3	120.8 (4)	C16—C15—H15	119.6
C7—C2—C1	118.6 (4)	C14—C15—H15	119.6
C7—C2—C3	120.5 (4)	C6—C5—H5	119.6
C2—C7—C6	117.7 (4)	C6—C5—C4	120.7 (5)
C2—C7—C8	117.9 (4)	C4—C5—H5	119.6
C8—C7—C6	124.3 (4)	C3—C4—C5	120.8 (5)
C10—C11—H11	119.2	C3—C4—H4	119.6
C10—C11—C12	121.5 (4)	C5—C4—H4	119.6
C12—C11—H11	119.2	C14—C13—C12	120.4 (5)
N2—C18—H18	119.1	C14—C13—H13	119.8
N2—C18—C17	121.7 (4)	C12—C13—H13	119.8

Symmetry codes: (i) −*x*+1, −*y*+1, −*z*+1; (ii) −*x*+1, −*y*+2, −*z*+1; (iii) −*x*+1, −*y*+1, −*z*; (iv) −*x*+1, −*y*+2, −*z*.

### Dibromidobis(isoquinoline N-oxide-κO)zinc(II) (II) Crystal data

**Table:**

[ZnBr_2_(C_9_H_7_NO)_2_]	*F*(000) = 1008
*M**_r_* = 515.50	*D*_x_ = 1.870 Mg m^−^^3^
Monoclinic, *C*2/*c*	Mo *K*α radiation, λ = 0.71073 Å
*a* = 17.1095 (10) Å	Cell parameters from 2412 reflections
*b* = 7.2020 (6) Å	θ = 2.5–33.1°
*c* = 14.9534 (11) Å	µ = 5.72 mm^−^^1^
β = 96.472 (6)°	*T* = 170 K
*V* = 1830.9 (2) Å^3^	Block, clear colourless
*Z* = 4	0.2 × 0.2 × 0.2 mm

### Dibromidobis(isoquinoline N-oxide-κO)zinc(II) (II) Data collection

**Table:**

XtaLAB Mini diffractometer	1671 independent reflections
Radiation source: fine-focus sealed X-ray tube, Enhance (Mo) X-ray Source	1402 reflections with *I* > 2σ(*I*)
Graphite monochromator	*R*_int_ = 0.044
ω scans	θ_max_ = 25.4°, θ_min_ = 2.4°
Absorption correction: multi-scan (CrysAlisPro; Rigaku OD, 2019)	*h* = −16→20
*T*_min_ = 0.358, *T*_max_ = 1.000	*k* = −8→8
2980 measured reflections	*l* = −18→17

### Dibromidobis(isoquinoline N-oxide-κO)zinc(II) (II) Refinement

**Table:**

Refinement on *F*^2^	Primary atom site location: dual
Least-squares matrix: full	Hydrogen site location: inferred from neighbouring sites
*R*[*F*^2^ > 2σ(*F*^2^)] = 0.052	H-atom parameters constrained
*wR*(*F*^2^) = 0.141	*w* = 1/[σ^2^(*F*_o_^2^) + (0.0927*P*)^2^] where *P* = (*F*_o_^2^ + 2*F*_c_^2^)/3
*S* = 1.03	(Δ/σ)_max_ < 0.001
1671 reflections	Δρ_max_ = 0.83 e Å^−^^3^
114 parameters	Δρ_min_ = −0.96 e Å^−^^3^
0 restraints	

### Dibromidobis(isoquinoline N-oxide-κO)zinc(II) (II) Special details

**Table:**

Geometry. All esds (except the esd in the dihedral angle between two l.s. planes) are estimated using the full covariance matrix. The cell esds are taken into account individually in the estimation of esds in distances, angles and torsion angles; correlations between esds in cell parameters are only used when they are defined by crystal symmetry. An approximate (isotropic) treatment of cell esds is used for estimating esds involving l.s. planes.

### Dibromidobis(isoquinoline N-oxide-κO)zinc(II) (II) Fractional atomic coordinates and isotropic or equivalent isotropic displacement parameters (Å^2^)

**Table:**

	*x*	*y*	*z*	*U*_iso_*/*U*_eq_	
Br1	0.60954 (3)	0.37505 (9)	0.21097 (4)	0.0370 (3)	
Zn1	0.500000	0.53998 (12)	0.250000	0.0276 (3)	
O1	0.5369 (2)	0.7150 (6)	0.3493 (3)	0.0362 (9)	
N1	0.4810 (2)	0.7741 (6)	0.3999 (3)	0.0293 (10)	
C2	0.3560 (3)	0.9088 (7)	0.4148 (4)	0.0274 (12)	
C7	0.3704 (3)	0.8846 (7)	0.5089 (4)	0.0306 (13)	
C1	0.4157 (3)	0.8517 (7)	0.3621 (4)	0.0293 (12)	
H1	0.408573	0.869682	0.298816	0.035*	
C3	0.2848 (3)	0.9829 (8)	0.3750 (5)	0.0357 (13)	
H3	0.275439	0.998479	0.311593	0.043*	
C9	0.4960 (3)	0.7503 (8)	0.4919 (4)	0.0342 (13)	
H9	0.544037	0.695474	0.517045	0.041*	
C4	0.2289 (3)	1.0325 (8)	0.4292 (5)	0.0393 (15)	
H4	0.180142	1.081746	0.403055	0.047*	
C8	0.4426 (3)	0.8049 (7)	0.5454 (4)	0.0325 (12)	
H8	0.453412	0.790004	0.608739	0.039*	
C6	0.3108 (4)	0.9413 (8)	0.5627 (5)	0.0383 (14)	
H6	0.319011	0.929530	0.626322	0.046*	
C5	0.2426 (4)	1.0119 (8)	0.5222 (5)	0.0443 (17)	
H5	0.202956	1.048433	0.558206	0.053*	

### Dibromidobis(isoquinoline N-oxide-κO)zinc(II) (II) Atomic displacement parameters (Å^2^)

**Table:**

	*U*^11^	*U*^22^	*U*^33^	*U*^12^	*U*^13^	*U*^23^
Br1	0.0276 (4)	0.0536 (4)	0.0301 (4)	0.0097 (2)	0.0041 (2)	−0.0043 (3)
Zn1	0.0200 (5)	0.0393 (5)	0.0237 (5)	0.000	0.0032 (3)	0.000
O1	0.0189 (18)	0.053 (2)	0.037 (2)	0.0036 (17)	0.0060 (15)	−0.013 (2)
N1	0.021 (2)	0.037 (2)	0.030 (3)	−0.0015 (18)	0.0032 (17)	−0.008 (2)
C2	0.023 (3)	0.031 (3)	0.028 (3)	−0.002 (2)	0.000 (2)	0.003 (2)
C7	0.026 (3)	0.035 (3)	0.031 (3)	−0.006 (2)	0.004 (2)	−0.004 (2)
C1	0.025 (3)	0.038 (3)	0.024 (3)	−0.004 (2)	−0.001 (2)	0.000 (2)
C3	0.026 (3)	0.038 (3)	0.042 (4)	−0.004 (2)	0.003 (2)	0.002 (3)
C9	0.027 (3)	0.042 (3)	0.032 (3)	−0.003 (2)	−0.004 (2)	0.003 (3)
C4	0.023 (3)	0.039 (3)	0.056 (4)	0.000 (2)	0.003 (3)	0.002 (3)
C8	0.038 (3)	0.033 (3)	0.024 (3)	−0.001 (2)	−0.005 (2)	0.004 (3)
C6	0.042 (4)	0.037 (3)	0.039 (4)	−0.007 (3)	0.015 (3)	−0.008 (3)
C5	0.028 (3)	0.043 (3)	0.065 (5)	−0.002 (2)	0.022 (3)	−0.011 (3)

### Dibromidobis(isoquinoline N-oxide-κO)zinc(II) (II) Geometric parameters (Å, º)

**Table:**

Zn1—Br1	2.3476 (7)	C1—H1	0.9500
Zn1—Br1^i^	2.3476 (7)	C3—H3	0.9500
Zn1—O1	1.995 (4)	C3—C4	1.369 (8)
Zn1—O1^i^	1.995 (4)	C9—H9	0.9500
O1—N1	1.353 (5)	C9—C8	1.340 (8)
N1—C1	1.317 (7)	C4—H4	0.9500
N1—C9	1.381 (8)	C4—C5	1.391 (10)
C2—C7	1.411 (8)	C8—H8	0.9500
C2—C1	1.421 (8)	C6—H6	0.9500
C2—C3	1.400 (8)	C6—C5	1.351 (9)
C7—C8	1.416 (8)	C5—H5	0.9500
C7—C6	1.428 (8)		
			
Br1—Zn1—Br1^i^	119.21 (5)	C2—C3—H3	120.7
O1—Zn1—Br1	108.06 (10)	C4—C3—C2	118.6 (6)
O1^i^—Zn1—Br1^i^	108.06 (10)	C4—C3—H3	120.7
O1—Zn1—Br1^i^	109.22 (11)	N1—C9—H9	120.1
O1^i^—Zn1—Br1	109.22 (11)	C8—C9—N1	119.7 (5)
O1—Zn1—O1^i^	101.6 (3)	C8—C9—H9	120.1
N1—O1—Zn1	115.5 (3)	C3—C4—H4	119.5
O1—N1—C9	117.0 (4)	C3—C4—C5	120.9 (6)
C1—N1—O1	120.8 (5)	C5—C4—H4	119.5
C1—N1—C9	122.2 (5)	C7—C8—H8	119.6
C7—C2—C1	117.4 (5)	C9—C8—C7	120.8 (6)
C3—C2—C7	121.2 (5)	C9—C8—H8	119.6
C3—C2—C1	121.4 (5)	C7—C6—H6	120.3
C2—C7—C8	118.8 (5)	C5—C6—C7	119.3 (6)
C2—C7—C6	118.1 (5)	C5—C6—H6	120.3
C8—C7—C6	123.1 (6)	C4—C5—H5	119.1
N1—C1—C2	120.9 (5)	C6—C5—C4	121.8 (6)
N1—C1—H1	119.5	C6—C5—H5	119.1
C2—C1—H1	119.5		
			
Zn1—O1—N1—C1	−54.2 (6)	C1—N1—C9—C8	0.9 (8)
Zn1—O1—N1—C9	126.2 (4)	C1—C2—C7—C8	−0.2 (7)
O1—N1—C1—C2	178.1 (4)	C1—C2—C7—C6	−180.0 (5)
O1—N1—C9—C8	−179.5 (5)	C1—C2—C3—C4	178.9 (5)
N1—C9—C8—C7	0.9 (8)	C3—C2—C7—C8	178.5 (5)
C2—C7—C8—C9	−1.2 (8)	C3—C2—C7—C6	−1.2 (8)
C2—C7—C6—C5	1.4 (8)	C3—C2—C1—N1	−176.8 (5)
C2—C3—C4—C5	0.7 (9)	C3—C4—C5—C6	−0.5 (10)
C7—C2—C1—N1	2.0 (8)	C9—N1—C1—C2	−2.4 (8)
C7—C2—C3—C4	0.2 (8)	C8—C7—C6—C5	−178.4 (6)
C7—C6—C5—C4	−0.6 (9)	C6—C7—C8—C9	178.6 (5)

Symmetry code: (i) −*x*+1, *y*, −*z*+1/2.

### Diiodidobis(isoquinoline N-oxide-κO)zinc(II) (III) Crystal data

**Table:**

[ZnI_2_(C_9_H_7_NO)_2_]	*Z* = 2
*M**_r_* = 609.48	*F*(000) = 576
Triclinic, *P*1	*D*_x_ = 2.080 Mg m^−^^3^
*a* = 7.7325 (3) Å	Mo *K*α radiation, λ = 0.71073 Å
*b* = 8.9596 (4) Å	Cell parameters from 6528 reflections
*c* = 14.8297 (9) Å	θ = 2.3–33.2°
α = 93.205 (4)°	µ = 4.45 mm^−^^1^
β = 99.873 (4)°	*T* = 170 K
γ = 104.852 (4)°	Irregular, clear colourless
*V* = 972.98 (9) Å^3^	0.32 × 0.13 × 0.06 mm

### Diiodidobis(isoquinoline N-oxide-κO)zinc(II) (III) Data collection

**Table:**

XtaLAB Mini diffractometer	3563 independent reflections
Radiation source: fine-focus sealed X-ray tube, Enhance (Mo) X-ray Source	3152 reflections with *I* > 2σ(*I*)
Graphite monochromator	*R*_int_ = 0.016
ω scans	θ_max_ = 25.4°, θ_min_ = 2.4°
Absorption correction: multi-scan (CrysAlisPro; Rigaku OD, 2019)	*h* = −9→9
*T*_min_ = 0.535, *T*_max_ = 1.000	*k* = −10→10
8495 measured reflections	*l* = −17→17

### Diiodidobis(isoquinoline N-oxide-κO)zinc(II) (III) Refinement

**Table:**

Refinement on *F*^2^	Primary atom site location: dual
Least-squares matrix: full	Hydrogen site location: inferred from neighbouring sites
*R*[*F*^2^ > 2σ(*F*^2^)] = 0.020	H-atom parameters constrained
*wR*(*F*^2^) = 0.048	*w* = 1/[σ^2^(*F*_o_^2^) + (0.0249*P*)^2^ + 0.3875*P*] where *P* = (*F*_o_^2^ + 2*F*_c_^2^)/3
*S* = 1.03	(Δ/σ)_max_ = 0.001
3563 reflections	Δρ_max_ = 0.59 e Å^−^^3^
226 parameters	Δρ_min_ = −0.39 e Å^−^^3^
0 restraints	

### Diiodidobis(isoquinoline N-oxide-κO)zinc(II) (III) Special details

**Table:**

Geometry. All esds (except the esd in the dihedral angle between two l.s. planes) are estimated using the full covariance matrix. The cell esds are taken into account individually in the estimation of esds in distances, angles and torsion angles; correlations between esds in cell parameters are only used when they are defined by crystal symmetry. An approximate (isotropic) treatment of cell esds is used for estimating esds involving l.s. planes.

### Diiodidobis(isoquinoline N-oxide-κO)zinc(II) (III) Fractional atomic coordinates and isotropic or equivalent isotropic displacement parameters (Å^2^)

**Table:**

	*x*	*y*	*z*	*U*_iso_*/*U*_eq_	
I2	1.04627 (3)	0.42553 (2)	0.19043 (2)	0.03413 (7)	
I1	0.93556 (3)	0.87866 (2)	0.26504 (2)	0.03405 (7)	
Zn1	0.84695 (4)	0.58158 (4)	0.24874 (2)	0.02607 (9)	
O1	0.5914 (3)	0.5206 (2)	0.17439 (15)	0.0331 (5)	
O2	0.8155 (3)	0.5066 (2)	0.37215 (14)	0.0324 (5)	
N1	0.5043 (3)	0.3742 (3)	0.13534 (16)	0.0242 (5)	
N2	0.7414 (3)	0.5881 (3)	0.42719 (16)	0.0261 (5)	
C2	0.4420 (3)	0.0990 (3)	0.12990 (19)	0.0242 (6)	
C1	0.5366 (4)	0.2500 (3)	0.17229 (19)	0.0252 (6)	
H1	0.623861	0.262763	0.227653	0.030*	
C7	0.3108 (4)	0.0815 (3)	0.04758 (19)	0.0251 (6)	
C9	0.3765 (4)	0.3613 (3)	0.05564 (19)	0.0269 (6)	
H9	0.354796	0.452253	0.031520	0.032*	
C11	0.5072 (4)	0.7042 (3)	0.45488 (19)	0.0240 (6)	
C16	0.6055 (4)	0.7592 (3)	0.54600 (19)	0.0251 (6)	
C8	0.2815 (4)	0.2191 (3)	0.01155 (19)	0.0280 (6)	
H8	0.195178	0.211549	−0.043598	0.034*	
C18	0.8379 (4)	0.6377 (3)	0.5163 (2)	0.0276 (6)	
H18	0.949142	0.612186	0.536568	0.033*	
C17	0.7732 (4)	0.7228 (3)	0.5744 (2)	0.0280 (6)	
H17	0.841205	0.758417	0.634815	0.034*	
C15	0.5324 (4)	0.8477 (3)	0.6039 (2)	0.0296 (6)	
H15	0.597063	0.886817	0.664503	0.036*	
C3	0.4758 (4)	−0.0347 (3)	0.1682 (2)	0.0303 (6)	
H3	0.564660	−0.023736	0.222759	0.036*	
C12	0.3390 (4)	0.7373 (3)	0.4237 (2)	0.0303 (7)	
H12	0.273178	0.700892	0.362968	0.036*	
C6	0.2151 (4)	−0.0703 (3)	0.0054 (2)	0.0325 (7)	
H6	0.127647	−0.084025	−0.049963	0.039*	
C10	0.5825 (4)	0.6174 (3)	0.3969 (2)	0.0277 (6)	
H10	0.518886	0.579961	0.335836	0.033*	
C14	0.3678 (4)	0.8771 (3)	0.5723 (2)	0.0327 (7)	
H14	0.318301	0.935043	0.611757	0.039*	
C5	0.2493 (4)	−0.1964 (4)	0.0450 (2)	0.0353 (7)	
H5	0.183619	−0.297723	0.017024	0.042*	
C4	0.3804 (4)	−0.1793 (3)	0.1265 (2)	0.0345 (7)	
H4	0.402405	−0.268730	0.152616	0.041*	
C13	0.2717 (4)	0.8220 (3)	0.4818 (2)	0.0322 (7)	
H13	0.158657	0.844158	0.460923	0.039*	

### Diiodidobis(isoquinoline N-oxide-κO)zinc(II) (III) Atomic displacement parameters (Å^2^)

**Table:**

	*U*^11^	*U*^22^	*U*^33^	*U*^12^	*U*^13^	*U*^23^
I2	0.03348 (11)	0.03541 (12)	0.03325 (12)	0.00911 (9)	0.00922 (9)	−0.00629 (8)
I1	0.03325 (11)	0.02439 (11)	0.03826 (12)	0.00528 (8)	−0.00469 (9)	−0.00142 (8)
Zn1	0.02493 (17)	0.02467 (17)	0.02604 (18)	0.00437 (13)	0.00288 (14)	−0.00172 (13)
O1	0.0277 (10)	0.0225 (10)	0.0411 (13)	0.0019 (8)	−0.0047 (9)	−0.0058 (9)
O2	0.0413 (12)	0.0327 (11)	0.0313 (11)	0.0188 (9)	0.0145 (10)	0.0053 (9)
N1	0.0209 (11)	0.0216 (12)	0.0275 (13)	0.0023 (9)	0.0039 (10)	−0.0003 (10)
N2	0.0294 (13)	0.0226 (12)	0.0280 (13)	0.0066 (10)	0.0096 (11)	0.0057 (10)
C2	0.0200 (13)	0.0278 (15)	0.0253 (15)	0.0062 (11)	0.0068 (11)	0.0003 (12)
C1	0.0215 (13)	0.0285 (15)	0.0231 (14)	0.0045 (11)	0.0013 (12)	0.0026 (12)
C7	0.0227 (13)	0.0289 (15)	0.0223 (14)	0.0047 (11)	0.0050 (12)	−0.0023 (11)
C9	0.0246 (14)	0.0315 (16)	0.0254 (15)	0.0085 (12)	0.0045 (12)	0.0067 (12)
C11	0.0243 (14)	0.0213 (14)	0.0244 (15)	0.0024 (11)	0.0044 (12)	0.0038 (11)
C16	0.0230 (14)	0.0213 (14)	0.0287 (15)	0.0006 (11)	0.0063 (12)	0.0046 (11)
C8	0.0240 (14)	0.0362 (17)	0.0208 (14)	0.0053 (12)	0.0010 (12)	0.0020 (12)
C18	0.0243 (14)	0.0282 (15)	0.0296 (16)	0.0056 (12)	0.0040 (12)	0.0068 (12)
C17	0.0257 (14)	0.0281 (16)	0.0241 (15)	0.0007 (12)	−0.0020 (12)	0.0018 (12)
C15	0.0324 (16)	0.0294 (16)	0.0242 (15)	0.0018 (12)	0.0084 (13)	0.0016 (12)
C3	0.0298 (15)	0.0335 (17)	0.0282 (16)	0.0098 (13)	0.0045 (13)	0.0041 (13)
C12	0.0261 (15)	0.0293 (16)	0.0303 (16)	0.0023 (12)	−0.0006 (13)	0.0028 (12)
C6	0.0303 (16)	0.0330 (17)	0.0294 (16)	0.0031 (13)	0.0035 (13)	−0.0050 (13)
C10	0.0279 (15)	0.0270 (15)	0.0262 (15)	0.0050 (12)	0.0030 (12)	0.0040 (12)
C14	0.0330 (16)	0.0327 (17)	0.0355 (17)	0.0084 (13)	0.0155 (14)	0.0033 (13)
C5	0.0357 (17)	0.0278 (16)	0.0368 (18)	0.0015 (13)	0.0055 (14)	−0.0061 (13)
C4	0.0390 (17)	0.0294 (17)	0.0379 (18)	0.0112 (13)	0.0114 (15)	0.0055 (13)
C13	0.0241 (15)	0.0352 (17)	0.0383 (18)	0.0075 (13)	0.0081 (13)	0.0087 (13)

### Diiodidobis(isoquinoline N-oxide-κO)zinc(II) (III) Geometric parameters (Å, º)

**Table:**

I2—Zn1	2.5504 (4)	C16—C17	1.418 (4)
I1—Zn1	2.5591 (4)	C16—C15	1.416 (4)
Zn1—O1	2.0130 (19)	C8—H8	0.9500
Zn1—O2	2.016 (2)	C18—H18	0.9500
O1—N1	1.356 (3)	C18—C17	1.358 (4)
O2—N2	1.355 (3)	C17—H17	0.9500
N1—C1	1.328 (3)	C15—H15	0.9500
N1—C9	1.383 (4)	C15—C14	1.374 (4)
N2—C18	1.388 (4)	C3—H3	0.9500
N2—C10	1.331 (4)	C3—C4	1.367 (4)
C2—C1	1.415 (4)	C12—H12	0.9500
C2—C7	1.420 (4)	C12—C13	1.368 (4)
C2—C3	1.418 (4)	C6—H6	0.9500
C1—H1	0.9500	C6—C5	1.366 (4)
C7—C8	1.426 (4)	C10—H10	0.9500
C7—C6	1.421 (4)	C14—H14	0.9500
C9—H9	0.9500	C14—C13	1.412 (4)
C9—C8	1.361 (4)	C5—H5	0.9500
C11—C16	1.424 (4)	C5—C4	1.412 (4)
C11—C12	1.414 (4)	C4—H4	0.9500
C11—C10	1.418 (4)	C13—H13	0.9500
			
I2—Zn1—I1	122.378 (14)	C9—C8—H8	119.8
O1—Zn1—I2	112.15 (6)	N2—C18—H18	120.0
O1—Zn1—I1	104.01 (6)	C17—C18—N2	119.9 (3)
O1—Zn1—O2	103.62 (9)	C17—C18—H18	120.0
O2—Zn1—I2	104.06 (6)	C16—C17—H17	119.6
O2—Zn1—I1	109.19 (6)	C18—C17—C16	120.9 (3)
N1—O1—Zn1	123.46 (16)	C18—C17—H17	119.6
N2—O2—Zn1	117.46 (15)	C16—C15—H15	120.0
O1—N1—C9	116.1 (2)	C14—C15—C16	120.0 (3)
C1—N1—O1	122.2 (2)	C14—C15—H15	120.0
C1—N1—C9	121.7 (2)	C2—C3—H3	120.0
O2—N2—C18	117.0 (2)	C4—C3—C2	120.0 (3)
C10—N2—O2	121.1 (2)	C4—C3—H3	120.0
C10—N2—C18	121.9 (2)	C11—C12—H12	120.2
C1—C2—C7	119.2 (3)	C13—C12—C11	119.5 (3)
C1—C2—C3	121.2 (3)	C13—C12—H12	120.2
C3—C2—C7	119.6 (3)	C7—C6—H6	120.2
N1—C1—C2	120.5 (3)	C5—C6—C7	119.7 (3)
N1—C1—H1	119.7	C5—C6—H6	120.2
C2—C1—H1	119.7	N2—C10—C11	120.6 (3)
C2—C7—C8	117.7 (2)	N2—C10—H10	119.7
C2—C7—C6	119.1 (3)	C11—C10—H10	119.7
C6—C7—C8	123.2 (3)	C15—C14—H14	119.7
N1—C9—H9	119.8	C15—C14—C13	120.6 (3)
C8—C9—N1	120.5 (3)	C13—C14—H14	119.7
C8—C9—H9	119.8	C6—C5—H5	119.3
C12—C11—C16	120.0 (3)	C6—C5—C4	121.3 (3)
C12—C11—C10	121.4 (3)	C4—C5—H5	119.3
C10—C11—C16	118.6 (3)	C3—C4—C5	120.3 (3)
C17—C16—C11	118.1 (3)	C3—C4—H4	119.8
C15—C16—C11	118.9 (3)	C5—C4—H4	119.8
C15—C16—C17	123.1 (3)	C12—C13—C14	121.0 (3)
C7—C8—H8	119.8	C12—C13—H13	119.5
C9—C8—C7	120.4 (3)	C14—C13—H13	119.5
			
Zn1—O1—N1—C1	30.8 (3)	C11—C16—C15—C14	1.0 (4)
Zn1—O1—N1—C9	−150.61 (19)	C11—C12—C13—C14	0.1 (4)
Zn1—O2—N2—C18	−127.1 (2)	C16—C11—C12—C13	−0.1 (4)
Zn1—O2—N2—C10	53.5 (3)	C16—C11—C10—N2	0.1 (4)
O1—N1—C1—C2	179.5 (2)	C16—C15—C14—C13	−1.1 (4)
O1—N1—C9—C8	−179.8 (2)	C8—C7—C6—C5	−179.1 (3)
O2—N2—C18—C17	178.6 (2)	C18—N2—C10—C11	1.2 (4)
O2—N2—C10—C11	−179.4 (2)	C17—C16—C15—C14	−179.2 (3)
N1—C9—C8—C7	0.8 (4)	C15—C16—C17—C18	−179.9 (3)
N2—C18—C17—C16	1.4 (4)	C15—C14—C13—C12	0.5 (4)
C2—C7—C8—C9	−0.3 (4)	C3—C2—C1—N1	179.6 (2)
C2—C7—C6—C5	0.6 (4)	C3—C2—C7—C8	−179.9 (2)
C2—C3—C4—C5	0.6 (4)	C3—C2—C7—C6	0.3 (4)
C1—N1—C9—C8	−1.2 (4)	C12—C11—C16—C17	179.8 (2)
C1—C2—C7—C8	0.2 (4)	C12—C11—C16—C15	−0.4 (4)
C1—C2—C7—C6	−179.6 (2)	C12—C11—C10—N2	179.7 (2)
C1—C2—C3—C4	179.0 (3)	C6—C7—C8—C9	179.4 (3)
C7—C2—C1—N1	−0.5 (4)	C6—C5—C4—C3	0.4 (5)
C7—C2—C3—C4	−0.9 (4)	C10—N2—C18—C17	−2.0 (4)
C7—C6—C5—C4	−0.9 (5)	C10—C11—C16—C17	−0.7 (4)
C9—N1—C1—C2	1.0 (4)	C10—C11—C16—C15	179.1 (2)
C11—C16—C17—C18	−0.1 (4)	C10—C11—C12—C13	−179.7 (3)

### Hexakis(isoquinoline N-oxide-κO)zinc(II) bis(perchlorate) (IV) Crystal data

**Table:**

[Zn(C_9_H_7_NO)_6_](ClO_4_)_2_	*D*_x_ = 1.495 Mg m^−^^3^
*M**_r_* = 1135.20	Mo *K*α radiation, λ = 0.71073 Å
Trigonal, *R*3	Cell parameters from 13112 reflections
*a* = 12.8217 (4) Å	θ = 2.4–33.0°
*c* = 26.5684 (9) Å	µ = 0.67 mm^−^^1^
*V* = 3782.6 (3) Å^3^	*T* = 170 K
*Z* = 3	Prism, clear colourless
*F*(000) = 1752	0.2 × 0.2 × 0.15 mm

### Hexakis(isoquinoline N-oxide-κO)zinc(II) bis(perchlorate) (IV) Data collection

**Table:**

XtaLAB Mini diffractometer	1545 independent reflections
Radiation source: fine-focus sealed X-ray tube, Enhance (Mo) X-ray Source	1450 reflections with *I* > 2σ(*I*)
Graphite monochromator	*R*_int_ = 0.017
ω scans	θ_max_ = 25.3°, θ_min_ = 2.0°
Absorption correction: multi-scan (CrysAlisPro; Rigaku OD, 2019)	*h* = −15→15
*T*_min_ = 0.914, *T*_max_ = 1.000	*k* = −15→15
11238 measured reflections	*l* = −31→31

### Hexakis(isoquinoline N-oxide-κO)zinc(II) bis(perchlorate) (IV) Refinement

**Table:**

Refinement on *F*^2^	Primary atom site location: dual
Least-squares matrix: full	Hydrogen site location: inferred from neighbouring sites
*R*[*F*^2^ > 2σ(*F*^2^)] = 0.028	H-atom parameters constrained
*wR*(*F*^2^) = 0.077	*w* = 1/[σ^2^(*F*_o_^2^) + (0.0393*P*)^2^ + 4.8988*P*] where *P* = (*F*_o_^2^ + 2*F*_c_^2^)/3
*S* = 1.06	(Δ/σ)_max_ < 0.001
1545 reflections	Δρ_max_ = 0.30 e Å^−^^3^
117 parameters	Δρ_min_ = −0.25 e Å^−^^3^
0 restraints	

### Hexakis(isoquinoline N-oxide-κO)zinc(II) bis(perchlorate) (IV) Special details

**Table:**

Geometry. All esds (except the esd in the dihedral angle between two l.s. planes) are estimated using the full covariance matrix. The cell esds are taken into account individually in the estimation of esds in distances, angles and torsion angles; correlations between esds in cell parameters are only used when they are defined by crystal symmetry. An approximate (isotropic) treatment of cell esds is used for estimating esds involving l.s. planes.

### Hexakis(isoquinoline N-oxide-κO)zinc(II) bis(perchlorate) (IV) Fractional atomic coordinates and isotropic or equivalent isotropic displacement parameters (Å^2^)

**Table:**

	*x*	*y*	*z*	*U*_iso_*/*U*_eq_	
C1	0.57812 (14)	0.42465 (14)	0.46049 (6)	0.0306 (3)	
H1	0.516846	0.343592	0.466025	0.037*	
N1	0.61267 (12)	0.46273 (12)	0.41383 (5)	0.0307 (3)	
O1	0.56235 (10)	0.38624 (11)	0.37552 (4)	0.0362 (3)	
Zn1	0.666667	0.333333	0.333333	0.03070 (15)	
C2	0.63025 (14)	0.50170 (15)	0.50200 (6)	0.0319 (4)	
C3	0.59763 (17)	0.45964 (18)	0.55216 (6)	0.0405 (4)	
H3	0.538797	0.378022	0.558427	0.049*	
C4	0.6517 (2)	0.5376 (2)	0.59144 (7)	0.0517 (5)	
H4	0.630487	0.509631	0.625046	0.062*	
C5	0.7383 (2)	0.6590 (2)	0.58245 (8)	0.0561 (6)	
H5	0.774576	0.711886	0.610150	0.067*	
C6	0.77093 (18)	0.70187 (19)	0.53467 (8)	0.0489 (5)	
H6	0.829315	0.784090	0.529361	0.059*	
C7	0.71806 (15)	0.62420 (15)	0.49288 (7)	0.0363 (4)	
C8	0.74836 (16)	0.66040 (15)	0.44206 (7)	0.0393 (4)	
H8	0.805433	0.741987	0.434702	0.047*	
C9	0.69756 (16)	0.58101 (15)	0.40386 (7)	0.0367 (4)	
H9	0.720339	0.606689	0.370095	0.044*	
Cl1	1.000000	1.000000	0.38486 (3)	0.03933 (19)	
O2	1.000000	1.000000	0.43897 (9)	0.0641 (7)	
O3	0.9353 (2)	0.88066 (16)	0.36730 (7)	0.1013 (8)	

### Hexakis(isoquinoline N-oxide-κO)zinc(II) bis(perchlorate) (IV) Atomic displacement parameters (Å^2^)

**Table:**

	*U*^11^	*U*^22^	*U*^33^	*U*^12^	*U*^13^	*U*^23^
C1	0.0301 (8)	0.0300 (8)	0.0354 (8)	0.0177 (7)	0.0042 (6)	0.0021 (6)
N1	0.0319 (7)	0.0323 (7)	0.0308 (7)	0.0183 (6)	0.0013 (5)	−0.0015 (5)
O1	0.0361 (6)	0.0409 (7)	0.0315 (6)	0.0191 (5)	−0.0016 (5)	−0.0074 (5)
Zn1	0.03368 (18)	0.03368 (18)	0.0248 (2)	0.01684 (9)	0.000	0.000
C2	0.0352 (8)	0.0367 (9)	0.0333 (8)	0.0251 (7)	0.0012 (6)	−0.0008 (7)
C3	0.0509 (10)	0.0499 (10)	0.0355 (9)	0.0364 (9)	0.0041 (8)	0.0028 (8)
C4	0.0705 (14)	0.0764 (15)	0.0340 (9)	0.0560 (13)	−0.0039 (9)	−0.0049 (9)
C5	0.0670 (14)	0.0700 (14)	0.0467 (11)	0.0457 (12)	−0.0180 (10)	−0.0249 (10)
C6	0.0466 (11)	0.0447 (11)	0.0599 (13)	0.0263 (9)	−0.0103 (9)	−0.0183 (9)
C7	0.0356 (9)	0.0357 (9)	0.0439 (10)	0.0226 (7)	−0.0020 (7)	−0.0059 (7)
C8	0.0379 (9)	0.0289 (8)	0.0506 (10)	0.0163 (7)	0.0052 (8)	0.0022 (7)
C9	0.0395 (9)	0.0342 (9)	0.0383 (9)	0.0198 (8)	0.0081 (7)	0.0072 (7)
Cl1	0.0419 (3)	0.0419 (3)	0.0343 (4)	0.02093 (13)	0.000	0.000
O2	0.0798 (12)	0.0798 (12)	0.0326 (13)	0.0399 (6)	0.000	0.000
O3	0.148 (2)	0.0507 (10)	0.0655 (11)	0.0199 (11)	−0.0338 (12)	−0.0110 (8)

### Hexakis(isoquinoline N-oxide-κO)zinc(II) bis(perchlorate) (IV) Geometric parameters (Å, º)

**Table:**

C1—H1	0.9500	C5—H5	0.9500
C1—N1	1.325 (2)	C5—C6	1.363 (3)
C1—C2	1.407 (2)	C6—H6	0.9500
N1—O1	1.3346 (17)	C6—C7	1.417 (3)
N1—C9	1.380 (2)	C7—C8	1.418 (3)
O1—Zn1	2.1008 (11)	C8—H8	0.9500
C2—C3	1.420 (2)	C8—C9	1.352 (3)
C2—C7	1.423 (2)	C9—H9	0.9500
C3—H3	0.9500	Cl1—O2	1.438 (2)
C3—C4	1.370 (3)	Cl1—O3^i^	1.4063 (18)
C4—H4	0.9500	Cl1—O3^ii^	1.4062 (18)
C4—C5	1.408 (3)	Cl1—O3	1.4063 (18)
			
N1—C1—H1	119.3	C4—C3—C2	119.50 (19)
N1—C1—C2	121.39 (15)	C4—C3—H3	120.3
C2—C1—H1	119.3	C3—C4—H4	119.7
C1—N1—O1	119.54 (13)	C3—C4—C5	120.60 (19)
C1—N1—C9	121.29 (14)	C5—C4—H4	119.7
O1—N1—C9	119.15 (13)	C4—C5—H5	119.4
N1—O1—Zn1	119.49 (9)	C6—C5—C4	121.12 (18)
O1^iii^—Zn1—O1^iv^	85.82 (4)	C6—C5—H5	119.4
O1^v^—Zn1—O1^vi^	85.82 (4)	C5—C6—H6	119.9
O1^v^—Zn1—O1^iv^	180.0	C5—C6—C7	120.2 (2)
O1^vii^—Zn1—O1	180.00 (6)	C7—C6—H6	119.9
O1^vii^—Zn1—O1^iv^	85.82 (4)	C6—C7—C2	118.61 (17)
O1^iii^—Zn1—O1	85.82 (4)	C6—C7—C8	124.03 (17)
O1^iv^—Zn1—O1	94.18 (4)	C8—C7—C2	117.36 (15)
O1^vi^—Zn1—O1^iv^	94.18 (4)	C7—C8—H8	119.4
O1^vii^—Zn1—O1^v^	94.18 (4)	C9—C8—C7	121.24 (16)
O1^v^—Zn1—O1	85.82 (4)	C9—C8—H8	119.4
O1^vii^—Zn1—O1^iii^	94.18 (4)	N1—C9—H9	119.9
O1^vi^—Zn1—O1	94.18 (4)	C8—C9—N1	120.12 (16)
O1^v^—Zn1—O1^iii^	94.18 (4)	C8—C9—H9	119.9
O1^vii^—Zn1—O1^vi^	85.82 (4)	O3^ii^—Cl1—O2	109.37 (8)
O1^iii^—Zn1—O1^vi^	180.0	O3^i^—Cl1—O2	109.37 (8)
C1—C2—C3	121.52 (16)	O3—Cl1—O2	109.37 (8)
C1—C2—C7	118.53 (15)	O3—Cl1—O3^i^	109.58 (8)
C3—C2—C7	119.95 (16)	O3^ii^—Cl1—O3	109.58 (8)
C2—C3—H3	120.3	O3^ii^—Cl1—O3^i^	109.57 (8)
			
C1—N1—O1—Zn1	−112.28 (13)	C2—C7—C8—C9	−1.3 (2)
C1—N1—C9—C8	0.8 (2)	C3—C2—C7—C6	−0.2 (2)
C1—C2—C3—C4	179.35 (15)	C3—C2—C7—C8	178.97 (15)
C1—C2—C7—C6	−179.82 (15)	C3—C4—C5—C6	−0.2 (3)
C1—C2—C7—C8	−0.7 (2)	C4—C5—C6—C7	−0.2 (3)
N1—C1—C2—C3	−176.87 (15)	C5—C6—C7—C2	0.4 (3)
N1—C1—C2—C7	2.8 (2)	C5—C6—C7—C8	−178.66 (18)
O1—N1—C9—C8	179.02 (15)	C6—C7—C8—C9	177.79 (17)
C2—C1—N1—O1	178.91 (13)	C7—C2—C3—C4	−0.3 (2)
C2—C1—N1—C9	−2.9 (2)	C7—C8—C9—N1	1.3 (3)
C2—C3—C4—C5	0.5 (3)	C9—N1—O1—Zn1	69.45 (16)

Symmetry codes: (i) −*x*+*y*+1, −*x*+2, *z*; (ii) −*y*+2, *x*−*y*+1, *z*; (iii) *y*+1/3, −*x*+*y*+2/3, −*z*+2/3; (iv) −*x*+*y*+1, −*x*+1, *z*; (v) *x*−*y*+1/3, *x*−1/3, −*z*+2/3; (vi) −*y*+1, *x*−*y*, *z*; (vii) −*x*+4/3, −*y*+2/3, −*z*+2/3.

### Pentaaqua(isoquinoline N-oxide-κO)zinc(II) dinitrate–isoquinoline N-oxide (1/2) (V) Crystal data

**Table:**

[Zn(C_9_H_7_NO)(H_2_O)_5_](NO_3_)_2_·2C_9_H_7_NO	*Z* = 2
*M**_r_* = 714.94	*F*(000) = 740
Triclinic, *P*1	*D*_x_ = 1.553 Mg m^−^^3^
*a* = 9.7360 (9) Å	Mo *K*α radiation, λ = 0.71073 Å
*b* = 11.5910 (9) Å	Cell parameters from 5791 reflections
*c* = 14.6381 (10) Å	θ = 1.9–31.6°
α = 74.352 (6)°	µ = 0.88 mm^−^^1^
β = 74.620 (7)°	*T* = 170 K
γ = 81.633 (7)°	Plank, clear whiteish colourless
*V* = 1528.8 (2) Å^3^	0.7 × 0.2 × 0.04 mm

### Pentaaqua(isoquinoline N-oxide-κO)zinc(II) dinitrate–isoquinoline N-oxide (1/2) (V) Data collection

**Table:**

XtaLAB Mini diffractometer	5599 independent reflections
Radiation source: fine-focus sealed X-ray tube, Enhance (Mo) X-ray Source	4252 reflections with *I* > 2σ(*I*)
Graphite monochromator	*R*_int_ = 0.047
ω scans	θ_max_ = 25.4°, θ_min_ = 2.1°
Absorption correction: multi-scan (CrysAlisPro; Rigaku OD, 2019)	*h* = −11→11
*T*_min_ = 0.752, *T*_max_ = 1.000	*k* = −13→13
13628 measured reflections	*l* = −17→17

### Pentaaqua(isoquinoline N-oxide-κO)zinc(II) dinitrate–isoquinoline N-oxide (1/2) (V) Refinement

**Table:**

Refinement on *F*^2^	Primary atom site location: dual
Least-squares matrix: full	Hydrogen site location: mixed
*R*[*F*^2^ > 2σ(*F*^2^)] = 0.044	H atoms treated by a mixture of independent and constrained refinement
*wR*(*F*^2^) = 0.106	*w* = 1/[σ^2^(*F*_o_^2^) + (0.0476*P*)^2^] where *P* = (*F*_o_^2^ + 2*F*_c_^2^)/3
*S* = 1.04	(Δ/σ)_max_ < 0.001
5599 reflections	Δρ_max_ = 0.51 e Å^−^^3^
464 parameters	Δρ_min_ = −0.48 e Å^−^^3^
10 restraints	

### Pentaaqua(isoquinoline N-oxide-κO)zinc(II) dinitrate–isoquinoline N-oxide (1/2) (V) Special details

**Table:**

Geometry. All esds (except the esd in the dihedral angle between two l.s. planes) are estimated using the full covariance matrix. The cell esds are taken into account individually in the estimation of esds in distances, angles and torsion angles; correlations between esds in cell parameters are only used when they are defined by crystal symmetry. An approximate (isotropic) treatment of cell esds is used for estimating esds involving l.s. planes.

### Pentaaqua(isoquinoline N-oxide-κO)zinc(II) dinitrate–isoquinoline N-oxide (1/2) (V) Fractional atomic coordinates and isotropic or equivalent isotropic displacement parameters (Å^2^)

**Table:**

	*x*	*y*	*z*	*U*_iso_*/*U*_eq_	
Zn1	0.38957 (4)	0.27598 (3)	0.47321 (2)	0.02573 (12)	
O5	0.2972 (3)	0.1986 (2)	0.62110 (16)	0.0336 (5)	
O2	0.2233 (2)	−0.03662 (19)	0.45547 (14)	0.0317 (5)	
O8	0.5068 (3)	0.3482 (2)	0.32840 (17)	0.0335 (5)	
O6	0.4139 (3)	0.1109 (2)	0.44532 (17)	0.0320 (5)	
O1	0.1881 (2)	0.3151 (2)	0.43902 (14)	0.0329 (5)	
O3	0.2216 (2)	0.63601 (19)	0.43681 (15)	0.0315 (5)	
O7	0.5973 (3)	0.2419 (2)	0.5086 (2)	0.0403 (6)	
O4	0.3663 (3)	0.4362 (2)	0.50705 (18)	0.0371 (6)	
O9	0.6657 (2)	0.0528 (2)	0.32553 (16)	0.0426 (6)	
N2	0.1959 (3)	−0.0507 (2)	0.37417 (17)	0.0254 (6)	
O10	0.6236 (3)	0.1957 (2)	0.20239 (17)	0.0494 (7)	
N1	0.1475 (3)	0.2886 (2)	0.36717 (17)	0.0259 (6)	
N3	0.1843 (3)	0.6184 (2)	0.35969 (17)	0.0242 (6)	
O14	0.6846 (3)	0.5678 (2)	0.15635 (16)	0.0506 (7)	
N5	0.6688 (3)	0.6144 (3)	0.22466 (18)	0.0319 (6)	
O12	0.6239 (3)	0.5538 (2)	0.31066 (16)	0.0550 (8)	
N4	0.6547 (3)	0.0879 (3)	0.23757 (19)	0.0348 (7)	
O13	0.6984 (3)	0.7187 (2)	0.21242 (18)	0.0561 (7)	
C21	0.0981 (3)	0.5780 (3)	0.2055 (2)	0.0266 (7)	
C8	0.1928 (3)	0.2520 (3)	0.2088 (2)	0.0274 (7)	
C27	0.2819 (3)	0.6092 (3)	0.2792 (2)	0.0286 (7)	
H27	0.379243	0.616684	0.275509	0.034*	
C18	0.3008 (3)	−0.0639 (3)	0.2975 (2)	0.0296 (7)	
H18	0.396900	−0.061642	0.299782	0.036*	
C10	0.0545 (3)	−0.0545 (3)	0.3745 (2)	0.0302 (7)	
H10	−0.019054	−0.045432	0.430116	0.036*	
O11	0.6767 (4)	0.0133 (3)	0.1888 (2)	0.0847 (11)	
C26	0.2442 (3)	0.5885 (3)	0.1985 (2)	0.0263 (7)	
C12	0.1282 (3)	−0.0853 (3)	0.2112 (2)	0.0297 (7)	
C17	0.2723 (3)	−0.0813 (3)	0.2125 (2)	0.0286 (7)	
C19	0.0419 (3)	0.6089 (3)	0.3690 (2)	0.0287 (7)	
H19	−0.026227	0.616785	0.427344	0.034*	
C1	0.0028 (3)	0.2796 (3)	0.3813 (2)	0.0307 (7)	
H1	−0.061138	0.287856	0.441167	0.037*	
C3	0.0448 (3)	0.2453 (3)	0.2200 (2)	0.0299 (7)	
C9	0.2384 (3)	0.2747 (3)	0.2853 (2)	0.0290 (7)	
H9	0.337290	0.280238	0.277881	0.035*	
C11	0.0214 (3)	−0.0713 (3)	0.2949 (2)	0.0326 (8)	
H11	−0.075967	−0.073691	0.295508	0.039*	
C20	−0.0004 (3)	0.5883 (3)	0.2944 (2)	0.0297 (7)	
H20	−0.098808	0.580508	0.301656	0.036*	
C2	−0.0479 (4)	0.2589 (3)	0.3095 (2)	0.0340 (8)	
H2	−0.147556	0.253496	0.319718	0.041*	
C4	−0.0010 (4)	0.2278 (3)	0.1409 (2)	0.0374 (8)	
H4	−0.099689	0.223752	0.146368	0.045*	
C22	0.0597 (3)	0.5589 (3)	0.1248 (2)	0.0323 (7)	
H22	−0.037509	0.551670	0.128068	0.039*	
C23	0.1620 (4)	0.5507 (3)	0.0416 (2)	0.0373 (8)	
H23	0.135062	0.536953	−0.012121	0.045*	
C25	0.3475 (3)	0.5804 (3)	0.1117 (2)	0.0362 (8)	
H25	0.445364	0.587591	0.106691	0.043*	
C6	0.2425 (4)	0.2205 (3)	0.0467 (2)	0.0375 (8)	
H6	0.308229	0.210789	−0.012074	0.045*	
C16	0.3825 (4)	−0.0944 (3)	0.1304 (2)	0.0382 (8)	
H16	0.479439	−0.092808	0.131277	0.046*	
C13	0.0982 (4)	−0.1022 (3)	0.1265 (2)	0.0385 (8)	
H13	0.002163	−0.105780	0.124593	0.046*	
C5	0.0964 (4)	0.2167 (3)	0.0569 (2)	0.0391 (9)	
H5	0.064065	0.206086	0.004116	0.047*	
C7	0.2916 (4)	0.2382 (3)	0.1211 (2)	0.0334 (8)	
H7	0.390965	0.241266	0.113941	0.040*	
C24	0.3058 (4)	0.5622 (3)	0.0347 (2)	0.0397 (8)	
H24	0.375070	0.557385	−0.023941	0.048*	
C14	0.2070 (4)	−0.1135 (3)	0.0474 (2)	0.0422 (9)	
H14	0.185987	−0.124268	−0.009398	0.051*	
C15	0.3490 (4)	−0.1093 (3)	0.0494 (2)	0.0433 (9)	
H15	0.423278	−0.116850	−0.006267	0.052*	
H6A	0.487 (3)	0.088 (3)	0.410 (2)	0.057 (13)*	
H5A	0.295 (4)	0.233 (3)	0.665 (2)	0.046 (11)*	
H8A	0.556 (3)	0.307 (3)	0.291 (2)	0.055 (13)*	
H5B	0.313 (4)	0.1248 (18)	0.641 (3)	0.056 (13)*	
H6B	0.356 (3)	0.066 (3)	0.446 (2)	0.039 (10)*	
H7A	0.628 (4)	0.286 (3)	0.533 (2)	0.041 (11)*	
H8B	0.543 (4)	0.411 (2)	0.323 (3)	0.062 (13)*	
H7B	0.649 (4)	0.179 (2)	0.515 (3)	0.073 (15)*	
H4A	0.318 (4)	0.497 (3)	0.486 (3)	0.079 (16)*	
H4B	0.380 (4)	0.436 (4)	0.5592 (18)	0.062 (13)*	

### Pentaaqua(isoquinoline N-oxide-κO)zinc(II) dinitrate–isoquinoline N-oxide (1/2) (V) Atomic displacement parameters (Å^2^)

**Table:**

	*U*^11^	*U*^22^	*U*^33^	*U*^12^	*U*^13^	*U*^23^
Zn1	0.0289 (2)	0.0246 (2)	0.0272 (2)	−0.00353 (15)	−0.00874 (15)	−0.00943 (15)
O5	0.0441 (14)	0.0312 (14)	0.0257 (12)	−0.0052 (11)	−0.0049 (11)	−0.0094 (11)
O2	0.0361 (13)	0.0362 (13)	0.0286 (12)	−0.0091 (10)	−0.0067 (10)	−0.0154 (10)
O8	0.0376 (14)	0.0332 (14)	0.0310 (13)	−0.0127 (12)	−0.0019 (11)	−0.0109 (12)
O6	0.0265 (13)	0.0299 (13)	0.0437 (14)	−0.0074 (11)	−0.0004 (11)	−0.0207 (11)
O1	0.0291 (12)	0.0521 (14)	0.0277 (12)	0.0048 (10)	−0.0136 (10)	−0.0240 (11)
O3	0.0353 (12)	0.0362 (13)	0.0333 (12)	0.0011 (10)	−0.0177 (10)	−0.0184 (10)
O7	0.0365 (14)	0.0334 (15)	0.0669 (18)	0.0000 (12)	−0.0282 (13)	−0.0248 (14)
O4	0.0617 (17)	0.0267 (13)	0.0347 (14)	0.0051 (12)	−0.0296 (13)	−0.0138 (11)
O9	0.0489 (15)	0.0452 (15)	0.0289 (13)	0.0084 (12)	−0.0068 (11)	−0.0096 (11)
N2	0.0308 (14)	0.0208 (13)	0.0268 (14)	−0.0059 (11)	−0.0067 (11)	−0.0077 (11)
O10	0.0631 (18)	0.0388 (15)	0.0385 (14)	0.0056 (13)	−0.0096 (12)	−0.0038 (12)
N1	0.0270 (14)	0.0292 (14)	0.0253 (14)	0.0007 (11)	−0.0102 (11)	−0.0104 (11)
N3	0.0291 (14)	0.0204 (13)	0.0276 (14)	0.0007 (11)	−0.0121 (11)	−0.0096 (11)
O14	0.0637 (17)	0.0682 (18)	0.0293 (13)	−0.0233 (14)	−0.0041 (12)	−0.0247 (13)
N5	0.0268 (15)	0.0460 (18)	0.0272 (15)	−0.0063 (13)	−0.0118 (12)	−0.0089 (14)
O12	0.083 (2)	0.0668 (18)	0.0223 (13)	−0.0474 (16)	−0.0046 (13)	−0.0080 (12)
N4	0.0354 (16)	0.0355 (17)	0.0294 (16)	−0.0090 (13)	0.0006 (12)	−0.0058 (14)
O13	0.097 (2)	0.0361 (15)	0.0424 (15)	−0.0149 (14)	−0.0265 (15)	−0.0065 (12)
C21	0.0288 (17)	0.0226 (16)	0.0313 (17)	0.0012 (13)	−0.0132 (14)	−0.0070 (14)
C8	0.0389 (19)	0.0210 (16)	0.0245 (16)	−0.0027 (14)	−0.0121 (14)	−0.0046 (13)
C27	0.0242 (16)	0.0278 (17)	0.0367 (18)	0.0002 (13)	−0.0101 (14)	−0.0110 (15)
C18	0.0246 (17)	0.0276 (17)	0.0344 (18)	−0.0055 (13)	−0.0015 (14)	−0.0075 (14)
C10	0.0247 (17)	0.0311 (17)	0.0336 (18)	−0.0062 (14)	−0.0042 (14)	−0.0067 (15)
O11	0.168 (4)	0.0488 (18)	0.0441 (17)	−0.010 (2)	−0.028 (2)	−0.0203 (15)
C26	0.0245 (16)	0.0235 (16)	0.0329 (17)	0.0024 (13)	−0.0089 (13)	−0.0104 (14)
C12	0.0318 (18)	0.0245 (16)	0.0323 (18)	−0.0048 (14)	−0.0075 (14)	−0.0047 (14)
C17	0.0306 (17)	0.0220 (16)	0.0315 (17)	−0.0034 (14)	−0.0037 (14)	−0.0069 (14)
C19	0.0253 (17)	0.0304 (17)	0.0313 (17)	−0.0019 (14)	−0.0069 (14)	−0.0092 (14)
C1	0.0293 (17)	0.0373 (19)	0.0275 (17)	−0.0056 (15)	−0.0066 (14)	−0.0098 (15)
C3	0.0359 (18)	0.0279 (17)	0.0292 (17)	−0.0090 (14)	−0.0134 (15)	−0.0035 (14)
C9	0.0251 (16)	0.0349 (18)	0.0295 (17)	0.0009 (14)	−0.0094 (14)	−0.0111 (15)
C11	0.0219 (16)	0.0377 (19)	0.0390 (19)	−0.0019 (14)	−0.0085 (14)	−0.0096 (16)
C20	0.0246 (16)	0.0345 (18)	0.0337 (18)	−0.0035 (14)	−0.0094 (14)	−0.0115 (15)
C2	0.0335 (18)	0.0388 (19)	0.0330 (18)	−0.0101 (15)	−0.0115 (15)	−0.0068 (15)
C4	0.045 (2)	0.041 (2)	0.0338 (19)	−0.0122 (17)	−0.0184 (16)	−0.0090 (16)
C22	0.0318 (18)	0.0378 (19)	0.0324 (18)	0.0015 (15)	−0.0126 (15)	−0.0145 (15)
C23	0.047 (2)	0.039 (2)	0.0336 (19)	0.0067 (16)	−0.0180 (16)	−0.0176 (16)
C25	0.0308 (18)	0.0361 (19)	0.043 (2)	0.0015 (15)	−0.0074 (15)	−0.0161 (16)
C6	0.058 (2)	0.0306 (18)	0.0248 (17)	0.0026 (17)	−0.0122 (16)	−0.0096 (15)
C16	0.0348 (19)	0.039 (2)	0.0378 (19)	−0.0065 (16)	0.0015 (15)	−0.0118 (16)
C13	0.045 (2)	0.039 (2)	0.0347 (19)	−0.0059 (17)	−0.0138 (16)	−0.0092 (16)
C5	0.060 (2)	0.0361 (19)	0.0305 (18)	−0.0088 (18)	−0.0225 (18)	−0.0098 (16)
C7	0.0382 (19)	0.0356 (19)	0.0260 (17)	0.0022 (15)	−0.0086 (14)	−0.0082 (15)
C24	0.042 (2)	0.042 (2)	0.0340 (19)	0.0036 (17)	−0.0017 (16)	−0.0191 (17)
C14	0.061 (3)	0.040 (2)	0.0288 (19)	−0.0065 (18)	−0.0103 (17)	−0.0139 (16)
C15	0.056 (2)	0.042 (2)	0.0298 (19)	−0.0083 (18)	0.0004 (17)	−0.0133 (16)

### Pentaaqua(isoquinoline N-oxide-κO)zinc(II) dinitrate–isoquinoline N-oxide (1/2) (V) Geometric parameters (Å, º)

**Table:**

Zn1—O5	2.110 (2)	C18—C17	1.414 (4)
Zn1—O8	2.130 (2)	C10—H10	0.9500
Zn1—O6	2.030 (2)	C10—C11	1.355 (4)
Zn1—O1	2.1078 (19)	C26—C25	1.413 (4)
Zn1—O7	2.174 (2)	C12—C17	1.415 (4)
Zn1—O4	2.015 (2)	C12—C11	1.411 (4)
O5—H5A	0.833 (18)	C12—C13	1.415 (4)
O5—H5B	0.833 (18)	C17—C16	1.409 (4)
O2—N2	1.341 (3)	C19—H19	0.9500
O8—H8A	0.827 (18)	C19—C20	1.352 (4)
O8—H8B	0.832 (19)	C1—H1	0.9500
O6—H6A	0.820 (18)	C1—C2	1.361 (4)
O6—H6B	0.816 (18)	C3—C2	1.414 (4)
O1—N1	1.337 (3)	C3—C4	1.417 (4)
O3—N3	1.346 (3)	C9—H9	0.9500
O7—H7A	0.815 (18)	C11—H11	0.9500
O7—H7B	0.819 (19)	C20—H20	0.9500
O4—H4A	0.821 (19)	C2—H2	0.9500
O4—H4B	0.807 (18)	C4—H4	0.9500
O9—N4	1.270 (3)	C4—C5	1.363 (5)
N2—C18	1.328 (4)	C22—H22	0.9500
N2—C10	1.383 (4)	C22—C23	1.369 (4)
O10—N4	1.242 (3)	C23—H23	0.9500
N1—C1	1.384 (4)	C23—C24	1.400 (5)
N1—C9	1.321 (4)	C25—H25	0.9500
N3—C27	1.322 (4)	C25—C24	1.369 (4)
N3—C19	1.375 (4)	C6—H6	0.9500
O14—N5	1.225 (3)	C6—C5	1.396 (5)
N5—O12	1.261 (3)	C6—C7	1.372 (4)
N5—O13	1.239 (3)	C16—H16	0.9500
N4—O11	1.226 (3)	C16—C15	1.369 (5)
C21—C26	1.420 (4)	C13—H13	0.9500
C21—C20	1.420 (4)	C13—C14	1.368 (4)
C21—C22	1.408 (4)	C5—H5	0.9500
C8—C3	1.418 (4)	C7—H7	0.9500
C8—C9	1.406 (4)	C24—H24	0.9500
C8—C7	1.417 (4)	C14—H14	0.9500
C27—H27	0.9500	C14—C15	1.398 (5)
C27—C26	1.412 (4)	C15—H15	0.9500
C18—H18	0.9500		
			
*Cg*1···*Cg*4	3.839 (2)	*Cg*2···*Cg*8	3.969 (2)
*Cg*1···*Cg*7	3.893 (2)	*Cg*4···*Cg*7^i^	3.943 (2)
*Cg*1···*Cg*8	3.8500 (19)	*Cg*5···*Cg*7^i^	3.7374 (19)
*Cg*2···*Cg*4	3.6617 (19)	*Cg*5···*Cg*8^i^	3.968 (2)
*Cg*2···*Cg*5	3.823 (2)		
			
O5—Zn1—O8	173.16 (9)	C11—C12—C17	117.9 (3)
O5—Zn1—O7	88.42 (10)	C11—C12—C13	123.3 (3)
O8—Zn1—O7	84.75 (10)	C13—C12—C17	118.8 (3)
O6—Zn1—O5	89.30 (10)	C18—C17—C12	118.2 (3)
O6—Zn1—O8	90.14 (10)	C16—C17—C18	121.9 (3)
O6—Zn1—O1	92.38 (9)	C16—C17—C12	119.9 (3)
O6—Zn1—O7	89.52 (9)	N3—C19—H19	120.2
O1—Zn1—O5	91.94 (9)	C20—C19—N3	119.5 (3)
O1—Zn1—O8	94.90 (9)	C20—C19—H19	120.2
O1—Zn1—O7	178.06 (9)	N1—C1—H1	120.0
O4—Zn1—O5	88.47 (10)	C2—C1—N1	120.0 (3)
O4—Zn1—O8	91.93 (10)	C2—C1—H1	120.0
O4—Zn1—O6	177.42 (10)	C2—C3—C8	117.4 (3)
O4—Zn1—O1	88.98 (9)	C2—C3—C4	124.3 (3)
O4—Zn1—O7	89.13 (10)	C4—C3—C8	118.3 (3)
Zn1—O5—H5A	121 (2)	N1—C9—C8	121.9 (3)
Zn1—O5—H5B	117 (3)	N1—C9—H9	119.0
H5A—O5—H5B	110 (4)	C8—C9—H9	119.1
Zn1—O8—H8A	125 (3)	C10—C11—C12	121.5 (3)
Zn1—O8—H8B	113 (3)	C10—C11—H11	119.2
H8A—O8—H8B	113 (4)	C12—C11—H11	119.2
Zn1—O6—H6A	121 (3)	C21—C20—H20	119.1
Zn1—O6—H6B	132 (2)	C19—C20—C21	121.8 (3)
H6A—O6—H6B	103 (4)	C19—C20—H20	119.1
N1—O1—Zn1	128.04 (16)	C1—C2—C3	121.2 (3)
Zn1—O7—H7A	123 (2)	C1—C2—H2	119.4
Zn1—O7—H7B	129 (3)	C3—C2—H2	119.4
H7A—O7—H7B	106 (4)	C3—C4—H4	119.9
Zn1—O4—H4A	127 (3)	C5—C4—C3	120.1 (3)
Zn1—O4—H4B	116 (3)	C5—C4—H4	119.9
H4A—O4—H4B	112 (4)	C21—C22—H22	119.9
O2—N2—C10	117.3 (2)	C23—C22—C21	120.3 (3)
C18—N2—O2	121.3 (2)	C23—C22—H22	119.9
C18—N2—C10	121.3 (3)	C22—C23—H23	119.5
O1—N1—C1	116.4 (2)	C22—C23—C24	120.9 (3)
C9—N1—O1	122.7 (2)	C24—C23—H23	119.5
C9—N1—C1	120.8 (3)	C26—C25—H25	120.2
O3—N3—C19	117.3 (2)	C24—C25—C26	119.6 (3)
C27—N3—O3	120.8 (2)	C24—C25—H25	120.2
C27—N3—C19	121.9 (3)	C5—C6—H6	119.8
O14—N5—O12	119.3 (3)	C7—C6—H6	119.8
O14—N5—O13	122.2 (3)	C7—C6—C5	120.4 (3)
O13—N5—O12	118.5 (3)	C17—C16—H16	120.2
O10—N4—O9	120.2 (3)	C15—C16—C17	119.6 (3)
O11—N4—O9	118.4 (3)	C15—C16—H16	120.2
O11—N4—O10	121.4 (3)	C12—C13—H13	119.9
C20—C21—C26	117.1 (3)	C14—C13—C12	120.2 (3)
C22—C21—C26	118.6 (3)	C14—C13—H13	119.9
C22—C21—C20	124.3 (3)	C4—C5—C6	121.5 (3)
C9—C8—C3	118.7 (3)	C4—C5—H5	119.2
C9—C8—C7	121.1 (3)	C6—C5—H5	119.2
C7—C8—C3	120.2 (3)	C8—C7—H7	120.3
N3—C27—H27	119.4	C6—C7—C8	119.4 (3)
N3—C27—C26	121.2 (3)	C6—C7—H7	120.3
C26—C27—H27	119.4	C23—C24—H24	119.7
N2—C18—H18	119.3	C25—C24—C23	120.6 (3)
N2—C18—C17	121.4 (3)	C25—C24—H24	119.7
C17—C18—H18	119.3	C13—C14—H14	119.7
N2—C10—H10	120.2	C13—C14—C15	120.6 (3)
C11—C10—N2	119.6 (3)	C15—C14—H14	119.7
C11—C10—H10	120.2	C16—C15—C14	121.0 (3)
C27—C26—C21	118.5 (3)	C16—C15—H15	119.5
C27—C26—C25	121.6 (3)	C14—C15—H15	119.5
C25—C26—C21	119.9 (3)		
			
Zn1—O1—N1—C1	155.2 (2)	C17—C16—C15—C14	−0.9 (5)
Zn1—O1—N1—C9	−26.6 (4)	C19—N3—C27—C26	−0.3 (4)
O2—N2—C18—C17	178.6 (3)	C1—N1—C9—C8	0.9 (5)
O2—N2—C10—C11	−178.5 (3)	C3—C8—C9—N1	0.7 (5)
O1—N1—C1—C2	176.7 (3)	C3—C8—C7—C6	1.1 (5)
O1—N1—C9—C8	−177.2 (3)	C3—C4—C5—C6	0.9 (5)
O3—N3—C27—C26	178.8 (2)	C9—N1—C1—C2	−1.5 (5)
O3—N3—C19—C20	−178.4 (3)	C9—C8—C3—C2	−1.6 (4)
N2—C18—C17—C12	−0.5 (4)	C9—C8—C3—C4	177.2 (3)
N2—C18—C17—C16	179.4 (3)	C9—C8—C7—C6	−177.6 (3)
N2—C10—C11—C12	0.1 (5)	C11—C12—C17—C18	0.1 (4)
N1—C1—C2—C3	0.5 (5)	C11—C12—C17—C16	−179.7 (3)
N3—C27—C26—C21	0.2 (4)	C11—C12—C13—C14	179.1 (3)
N3—C27—C26—C25	179.0 (3)	C20—C21—C26—C27	−0.4 (4)
N3—C19—C20—C21	−1.0 (5)	C20—C21—C26—C25	−179.3 (3)
C21—C26—C25—C24	−0.1 (5)	C20—C21—C22—C23	179.7 (3)
C21—C22—C23—C24	−0.7 (5)	C2—C3—C4—C5	179.4 (3)
C8—C3—C2—C1	1.0 (5)	C4—C3—C2—C1	−177.7 (3)
C8—C3—C4—C5	0.6 (5)	C22—C21—C26—C27	179.2 (3)
C27—N3—C19—C20	0.7 (4)	C22—C21—C26—C25	0.3 (4)
C27—C26—C25—C24	−179.0 (3)	C22—C21—C20—C19	−178.7 (3)
C18—N2—C10—C11	−0.5 (4)	C22—C23—C24—C25	0.9 (5)
C18—C17—C16—C15	−179.0 (3)	C13—C12—C17—C18	179.8 (3)
C10—N2—C18—C17	0.6 (4)	C13—C12—C17—C16	−0.1 (5)
C26—C21—C20—C19	0.9 (4)	C13—C12—C11—C10	−179.6 (3)
C26—C21—C22—C23	0.1 (5)	C13—C14—C15—C16	0.3 (5)
C26—C25—C24—C23	−0.5 (5)	C5—C6—C7—C8	0.3 (5)
C12—C17—C16—C15	0.8 (5)	C7—C8—C3—C2	179.6 (3)
C12—C13—C14—C15	0.5 (5)	C7—C8—C3—C4	−1.6 (4)
C17—C12—C11—C10	0.0 (5)	C7—C8—C9—N1	179.5 (3)
C17—C12—C13—C14	−0.6 (5)	C7—C6—C5—C4	−1.4 (5)

Symmetry code: (i) *x*, *y*−1, *z*.

### Pentaaqua(isoquinoline N-oxide-κO)zinc(II) dinitrate–isoquinoline N-oxide (1/2) (V) Hydrogen-bond geometry (Å, º)

**Table:**

*D*—H···*A*	*D*—H	H···*A*	*D*···*A*	*D*—H···*A*
O6—H6*A*···O9	0.82 (2)	1.91 (2)	2.723 (3)	173 (4)
O5—H5*A*···O13^ii^	0.83 (2)	2.04 (2)	2.861 (3)	168 (3)
O8—H8*A*···O10	0.83 (2)	2.00 (2)	2.808 (3)	164 (4)
O5—H5*B*···O9^iii^	0.83 (2)	1.98 (2)	2.802 (3)	171 (4)
O6—H6*B*···O2	0.82 (2)	1.84 (2)	2.651 (3)	177 (3)
O7—H7*A*···O3^ii^	0.82 (2)	2.03 (2)	2.798 (3)	156 (3)
O8—H8*B*···O12	0.83 (2)	1.88 (2)	2.710 (3)	179 (4)
O7—H7*B*···O2^iii^	0.82 (2)	1.94 (2)	2.755 (3)	173 (4)
O4—H4*A*···O3	0.82 (2)	1.81 (2)	2.634 (3)	176 (5)
O4—H4*B*···O12^ii^	0.81 (2)	1.93 (2)	2.729 (3)	169 (4)

Symmetry codes: (ii) −*x*+1, −*y*+1, −*z*+1; (iii) −*x*+1, −*y*, −*z*+1.
